# METTL3-mediated m6A methylation on lncRNA H19 inhibits intrahepatic cholangiocarcinoma progression through PPARγ downregulation

**DOI:** 10.7150/ijbs.120413

**Published:** 2025-09-29

**Authors:** Rong Xiao, Xinya Lu, Fang Huang, Yaru Zhao, Hao Jin, Xiaoyuan Jia, Biao Huang, Yigang Wang, Liang Chu

**Affiliations:** 1 College of Life Sciences and Medicine, Zhejiang Sci-Tech University, Hangzhou 310018, China; Oncology Department, Zhejiang Sci-Tech University Shaoxing Academy of Biomedicine, Shaoxing 312366, China.; 2 Hepatic Surgery Center, Tongji Hospital, Tongji Medical College, Huazhong University of Science and Technology, Wuhan 430030, China.; 3 Cancer Center, Department of Pathology, Zhejiang Provincial People's Hospital, Affiliated People's Hospital, Hangzhou Medical College, Hangzhou 310014, China.

**Keywords:** ICCA, m6A modification, METTL3, H19, PPARγ, oncolytic adenovirus

## Abstract

Intrahepatic cholangiocarcinoma (ICCA), the second most prevalent primary liver malignancy, remains poorly understood at the molecular level. Research into the function of N6-methyladenosine (m6A) modification in the formation of ICCA and its potential as a therapeutic approach is being spurred by mounting evidence that it plays a crucial role in tumor biology. Immunohistochemical examination of patient samples in this investigation revealed a significant decrease in m6A methyltransferase METTL3 expression, accompanied by lower levels, which were associated with a lower overall survival rate. Functional assays demonstrated that the enforced expression of METTL3 inhibited ICCA cell proliferation and migration, while concurrently increasing the levels of the long non-coding RNA H19. Mechanistic experiments using RNA-binding protein immunoprecipitation and methylated RNA immunoprecipitation confirmed that METTL3 directly interacted with H19 and enhanced its m6A modification. Importantly, silencing of H19 reversed the growth- and migration-suppressive effects of METTL3, whereas H19 overexpression counteracted the phenotype induced by METTL3 downregulation. Further analysis revealed that the METTL3-H19 regulatory axis suppressed the expression of peroxisome proliferator-activated receptor gamma (PPARγ). Moreover, an oncolytic adenovirus engineered to overexpress H19, in combination with the PPARγ inhibitor BAY-4931, elicited potent antitumor effects both *in vitro* and *in vivo*. Collectively, these findings identify METTL3-mediated m6A modification of H19 as a critical suppressor of ICCA progression through modulation of PPARγ signaling. One interesting treatment option for ICCA may be the use of H19-armed oncolytic adenoviruses, especially when combined with PPARγ suppression.

## Introduction

Intrahepatic cholangiocarcinoma (ICCA) is an aggressive malignancy that originates from the epithelial lining of the intrahepatic bile ducts. Over the past decade, both its incidence and mortality have shown a concerning upward trend, with nearly half of the patients presenting without any identifiable risk factors 1,2. Currently, surgical resection offers the only potentially curative option; however, this approach is feasible in merely 20-30% of patients diagnosed at an early to intermediate stage. For those with unresectable disease, systemic therapy with gemcitabine combined with cisplatin remains the standard first-line regimen, but its clinical benefit is often modest and unsatisfactory 3,4. Recent advances in molecular profiling of ICCA have enabled the development of targeted therapeutic approaches, particularly agents directed against fibroblast growth factor receptor 2 (FGFR2) fusions and isocitrate dehydrogenase 1 (IDH1) mutations, which have shown encouraging outcomes in selected patient subsets 5-7. Despite these advances, the majority of ICCA patients still lack effective treatment options. This emphasizes the urgent need to identify new treatment targets that can enhance clinical care and patient survival, as well as to clarify the molecular mechanisms underlying ICCA development.

Recent studies on N6-methyladenosine (m6A) alteration have highlighted the molecular processes behind the development of cancerous tumors. In eukaryotes, m6A is the most common type of internal RNA modification. It is a dynamic and reversible process 8,9. The deposition of m6A marks is catalyzed by methyltransferases, collectively referred to as "writers," such as methyltransferase-like 3 (METTL3), methyltransferase-like 14 (METTL14), and Wilms tumor 1-associated protein (WTAP). However, "erasers," such as alkylation repair homolog protein 5 (ALKBH5) and fat mass and obesity-associated protein (FTO), can eliminate these alterations. Furthermore, certain RNA-binding proteins called "readers," like YTH domain family proteins (YTHDF1/2/3) and insulin-like growth factor 2 mRNA-binding proteins (IGF2BP1/2/3), mediate m6A functions by recognizing m6A motifs and controlling crucial RNA functions like translation, stability, and splicing 10,11. Dysregulation of these m6A modulators has been increasingly linked to tumor initiation, progression, metastasis, metabolic reprogramming, therapeutic resistance, and remodeling of the tumor microenvironment 8,9,12,13. Yet, the exact biological functions of m6A modification in ICCA, along with its underlying regulatory networks, remain largely uncharacterized.

The present research demonstrated that METTL3 suppressed ICCA cell proliferation and migration by promoting the expression of the long non-coding RNA (lncRNA) H19 in an m6A-dependent manner. The recognition of H19 by IGF2BP1 was found to be essential for this process. Functionally, enforced H19 expression attenuated peroxisome proliferator-activated receptor gamma (PPARγ) signaling, thereby restricting ICCA progression. Moreover, an engineered oncolytic adenovirus, SD55-H19, designed to overexpress H19, revealed notable antitumor activity against ICCA, underscoring its potential as a novel targeted therapeutic approach.

## Materials and Methods

### Patient tissue samples

58 paired ICCA tissues and identical neighboring normal bile duct tissues were collected from patients who had intrahepatic cholangiocarcinectomy at the Hepatic Surgery Center, Tongji Hospital, Huazhong University of Science and Technology (Wuhan, China) between January 2013 and June 2019. This patient cohort was designated as the TJ cohort. The diagnosis of ICCA in all cases was confirmed by histopathological evaluation, and complete clinicopathological information was collected for each patient. The 8th edition of the *American Joint Committee on Cancer* (AJCC) Cancer Staging Manual's criteria were used to establish tumor staging 14. Formalin-fixed paraffin-embedded (FFPE) tissues were prepared for tissue microarray development, and snap-frozen ICCA specimens were employed for RNA extraction in experimental studies. The period of time between surgical resection and either death or the final follow-up appointment was known as overall survival (OS). The experimental procedures were carried out following the ethical standards of the Declaration of Helsinki and were approved by the Institutional Review Board of Tongji Hospital (Approval No. TJ-IRB202409062). Written informed consent was obtained from every patient before participation in the study.

### Cell lines and experimental culture

Human ICCA cell lines HuCC-T1 (RCB1960) and RBE (RCB1292) were obtained from the RIKEN BioResource Research Center (Ibaraki, Japan; Supplementary Table 1) 15,16. The Chinese Academy of Sciences' Type Culture Collection (Shanghai, China) supplied the 293T (SCSP-502) and 293A (SCSP-5094) cell lines. The supplier of the Hibepic cell line (CP-H042) was Procell Life Science and Technology, located in Wuhan, China. HuCC-T1 and Hibepic cells were maintained in RPMI-1640 medium (C11875500BT, Gibco, Grand Island, USA) supplemented with 10% fetal bovine serum (FBS; FCS500, Excell, Suzhou, China). 10% FBS was added to DMEM media (C11995500BT, Gibco) for the culture of RBE, 293A, and 293T cells. Every cell line was incubated in a humidified environment with 5% CO₂ at 37°C.

### siRNA transfection

The siRNAs were developed using the web application siDirect (http://sidirect2.rnai.jp/) and transfected into cells using Lipofectamine 2000 following the manufacturer's instructions (11668027, Invitrogen, Waltham, USA). Eight hours after transfection, the replacement of the culture medium was carried out with fresh growth medium. The sequences of all the siRNAs are presented in Supplementary Table 2.

### Lentivirus production and infection

The full-length open reading frame (ORF) of human METTL3 or H19 was cloned into the pCDH-CMV-MCS-EF1-copGFP-T2A-Puro lentiviral vector. Short hairpin RNAs (shRNAs) targeting human METTL3 or H19 were designed using the online tool provided by Sigma-Aldrich (https://www.sigmaaldrich.cn), and the corresponding sequences are listed in Supplementary Table 2. Using Lipofectamine 2000 (Invitrogen, USA), the co-transfection of the transfer vector plasmid, pSPAX2, and pMD2.G was carried out into 293T cells at a 4:3:1 ratio to package the lentivirus. Fresh growth medium was added 6hr after transfection. Following the manufacturer's instructions, viral supernatants were collected 48 and 72hr after transfection, combined, and concentrated using a lentivirus concentration solution (C2901M, Beyotime, Beijing, China). Furthermore, the ICCA cells were infected with the produced lentivirus and then selected with puromycin (HY-B1743, MedChemExpress, Shanghai, China) for two weeks to create stable cell lines.

### RNA extraction and real-time quantitative PCR (RT-qPCR) assay

Total RNA was isolated from cultured cells or homogenized tissue samples using TRIzol reagent (GK3016, Generay, Shanghai, China) and quantified with a NanoDrop 2000 spectrophotometer. The HiFiScript cDNA Synthesis Kit (CW2569M, CWBIO, Taizhou, China) was used to reverse-transcribe 2μg of total RNA to synthesize complementary DNA (cDNA). Following the manufacturer's instructions, the RT-qPCR experiment was carried out on an Applied Biosystems 7500 Real-Time PCR System using SYBR qPCR Master Mix (Q311-02, Vazyme, Nanjing, China). Relative gene expression levels were determined using the 2^-ΔΔCt^ method, with GAPDH serving as the internal control. Primer sequences used in this study are provided in Supplementary Table 2.

### Western blot

After being cleaned with PBS, the cells were lysed in RIPA buffer (Beyotime, P0013B). To exclude debris, the lysates were centrifuged at 10,000×g for 5min at 4°C after being heated to 100°C for 10min. Protein concentrations were determined using a BCA Protein Assay Kit (23227, Thermo, Waltham, USA). Proteins in equal quantities were separated on 10% SDS-PAGE gels and then put onto PVDF membranes with 0.45μm (IPVH08100, Millipore, Bedford, USA). After blocking the membranes for 2hr with 5% non-fat milk and washing them three times with TBST, the membranes were incubated overnight with the proper primary antibodies at 4°C. Following washing, secondary antibodies were added to the membranes and incubated for 2hr at room temperature. Employing an ECL detection device (E411-04, Vazyme), target proteins were observed. The antibodies are listed in Supplementary Table 3.

### Cell proliferation assays

Cell proliferation was evaluated using 3-[4,5-dimethylthiazol-2-yl]-2,5 diphenyl tetrazolium bromide (MTT) and colony formation assays. Cells were seeded into 96-well plates at a density of 2000 cells per well, cultured for 24 to 96hr and then used for the MTT assay. Each well holding 100μL of culture media was then filled with 20μL of MTT solution. Following a 4-hour incubation period, the medium was discarded, and 150μL of Dimethyl sulfoxide (DMSO) was added. Plates were shaken for 10min at room temperature, and absorbance was measured at 490nm using a microplate reader.

For the colony formation assay, 500 cells per well were seeded into three duplicate wells of a 6-well plate. Every five days, the culture medium was changed. Colonies were preserved during a 2-week incubation period, and stained with crystal violet solution (1.5% crystal violet in 10% methanol). Colonies were photographed, and the quantity of visible colonies was quantified.

### Cell migration assays

Cell migratory capacity was assessed using transwell and wound healing assays. In the transwell test, 100μL of serum-free media was used to seed 1×10⁴ cells into the upper chamber of transwell inserts, and 500μL of medium supplemented with 20% FBS was introduced to the lower chamber as a chemoattractant. A cotton swab was used to carefully remove any non-migrated cells that remained on the upper surface following a 36-hour incubation period. Cells that had moved to the bottom surface were fixed, dyed with crystal violet, photographed, and counted.

To perform the wound healing experiment, 8×10⁵ cells were seeded into 6-well plates and cultured in the medium with 10% FBS until they fully adhered. A linear wound was created across the monolayer using a 200μL pipette tip guided along a straight ruler to ensure uniformity. Detached cells were removed by washing 3x with PBS, and fresh serum-free medium was added. The scratch gap was photographed at 0 and 48hr using a microscope, and the wound area was quantified with ImageJ software.

### Measurement of the IC_50_ Value

To assess the impact of BAY-4931 on cell viability, 3000 cells per well were seeded into 96-well plates and treated with the indicated concentrations of BAY-4931 for 48hr. Cell viability was then determined using the MTT assay, and the half-maximal inhibitory concentration (IC₅₀) was determined.

### RNA stability assay

After being seeded into 6-well plates and left to adhere overnight, the ICCA cells were treated for 0, 3, and 6hr with actinomycin D (5μg/mL; HY-17559, MedChemExpress, Shanghai, China). After that, total RNA was extracted using the TRIzol reagent and examined by RT-qPCR.

### Luciferase reporter assay

Potential m6A modification sites within H19 were predicted using the SRAMP online tool (http://www.cuilab.cn/sramp/). The full-length H19 sequence, or a mutant construct in which adenosine (A) residues within the predicted m6A motifs were substituted with cytosine (C), was cloned into the pGL3-control vector to generate dual-luciferase reporter plasmids. These plasmids were used to transfect ICCA cells seeded in 6-well plates, and the Luc-Pair Duo-Luciferase Assay Kit (LF003, GeneCopoeia, Rockville, USA) was used to lyse the cells 48hr later. Luciferase activity was then measured using the Luc-Pair Duo-Luciferase Assay System (GeneCopoeia) according to the manufacturer's guidelines.

### RNA immunoprecipitation (RIP) assay

The RIP assay was performed using the Magna RIP kit (17-700, Millipore) according to the manufacturer's instructions. In brief, cells were lysed with complete RIP lysis buffer, and the lysates were incubated with magnetic beads conjugated to specific antibodies in RIP immunoprecipitation buffer. After thorough washing with RIP wash buffer, the immunoprecipitated RNA was extracted using TRIzol reagent, analyzed by RT-qPCR, and normalized to the corresponding input. IgG was used as a negative control.

### m6A RNA methylation quantification

The EpiQuik m6A RNA Methylation Quantification Kit (P-9005-48, Epigentek, Farmingdale, USA) was used to measure global m6A levels according to the manufacturer's instructions. In brief, 200ng of RNA, mixed with Binding Solution, was added to each strip well and incubated at 37°C for 90min. After washing, the capture antibody, detection antibody, and enhancer solution were sequentially applied. Absorbance at 450nm was used to measure the m6A level, and a standard curve was used to calculate the relative m6A content.

### Methylated RNA immunoprecipitation (MeRIP)-qPCR assay

MeRIP assays were conducted using the Magna MeRIP^™^ m^6^A Kit (17-10499, Millipore) according to the manufacturer's instructions. Briefly, 300μg of total RNA was extracted, fragmented to ~100 nucleotides using RNA fragmentation reagent, and immunoprecipitated with 10μg of anti-m6A antibody or control mouse IgG conjugated to Magna ChIP Protein A/G Magnetic Beads. N6-Methyladenosine 5′-monophosphate sodium salt was used for elution. The enriched RNAs were then purified and subjected to RT-qPCR using the primers listed in Supplementary Table 2.

### Construction and production of oncolytic adenovirus

The adenoviral shuttle plasmid pSD55 (pShuttle carrying the E1 gene with a deletion of the E1B 55-kDa gene) and the packaging backbone plasmid pAdeasy-1 were maintained in our laboratory 17. The full-length H19 sequence was cloned into pSD55 to construct pSD55-H19, which was verified by DNA sequencing. The EGFP gene was used as a control. The oncolytic adenoviruses SD55-H19 and SD55-EGFP were generated in 293A cells following homologous recombination in *E. coli* BJ-5183. Specifically, pSD55-H19 or pSD55-EGFP was co-transfected with pAdeasy-1 into competent BJ-5183 strain to obtain recombinant adenoviral genomes. After Pac I digestion, the recombinant genomes were transfected into 293A cells using Lipofectamine 2000, and oncolytic adenoviruses were produced within 7-10 days. Cesium chloride gradient ultracentrifugation was used to purify the viruses after they were isolated by plaque purification and amplified in 293A cells. Viral titers were expressed as plaque-forming units (pfu)/mL and were measured while employing the tissue culture infectious dose 50% (TCID50) test in HEK293 cells.

### Cytotoxicity assay

Cells were seeded into 96-well plates at a density of 5×10³ cells per well, followed by infecting the cells with SD55-EGFP or SD55-H19 at different multiplicities of infection (MOI). After incubation at 37°C for the indicated time, cell viability was assessed using the MTT assay. The percentage of viable cells was calculated using the formula: Cell viability (%) = (OD₄₉₀ of virus-infected cells/OD₄₉₀ of mock-treated cells) ×100.

### Viral progeny assay

HuCC-T1, RBE, and Hibepic cells were seeded in 6-well plates at a density of 5×10^5^ cells per well and infected with the virus at an MOI of 1. Six hrs post-infection, the medium was removed, cells were rinsed twice with PBS, and fresh medium was added. At 24, 48, 72, and 96hr, both cells and supernatants were collected after three freeze-thaw cycles followed by centrifugation. Viral DNA was extracted, and the Ct value of the Hexon gene was determined by qPCR. Virus titers were calculated using a standard curve.

### Cytopathic assay

RBE and HuCC-T1 cells were cultured to roughly 50% confluence after being seeded in 24-well plates. After that, cells were cultured for 48 hrs at 37°C after being infected with SD55-EGFP or SD55-H19 at different MOIs. After removing the culture medium, the cells were stained for 30 minutes using a crystal violet solution. Plates were photographed, and images from each replicate were analyzed and quantified using ImageJ software.

### Animal experiments

Six-week-old female NOD/SCID mice (T001492, GemPharmatech, Jiangsu, China) were subcutaneously injected with a total of 1×10^7^ HuCC-T1 cells suspended in 200μL PBS/Matrigel mixture (1:1) 16,18-20. Mice were divided into four groups (n=8) at random once the tumors had reached a volume of approximately 100-120mm³. Three groups received daily intratumoral injections (100μL) of PBS, SD55-EGFP (5×10⁸PFU), or SD55-H19 (5×10⁸PFU), respectively, combined with oral gavage of DMSO. The fourth group was treated with intratumoral injection of SD55-H19 (5×10⁸PFU/100μL) together with oral administration of BAY-4931 (30mg/kg; HY-148352, MedChemExpress). Treatments were administered once daily for four consecutive days. Tumor size was measured twice weekly using a vernier caliper, and volume was determined with the formula: volume = 0.52 × length × width². Twenty-eight days after the initial treatment, mice were euthanized. Tumors were excised for hematoxylin and eosin (HE) staining, and Ki67 expression was assessed by immunohistochemistry (IHC). All procedures were conducted in accordance with the *Guide for the Care and Use of Laboratory Animals* and approved by the Committee on the Ethics of Animal Experiments of Zhejiang Sci-Tech University (20240201-05).

### IHC staining

The IHC staining for METTL3 and PPARγ was performed on a tissue microarray (TMA). After deparaffinization, rehydration, and application of EDTA buffer for antigen retrieval, slides were incubated with primary antibodies against PPARγ (Rabbit polyclonal antibody, A11183, ABclonal) and METTL3 (Rabbit polyclonal antibody, A8370, ABclonal, Wuhan, China). Staining intensity was classified into four grades (intensity scores): negative (0), weak 1, moderate 2, and strong 3. The percentage of positive cells was scored on a scale of 0 to 100%. The calculation of the histological score (H-score) was then carried out while employing the formula: H-score = percentage score × intensity score × 100, yielding a composite range of 0-300. IHC scores were independently evaluated by three pathologists blinded to patient information, as described earlier 21.

### Online bioinformatic analysis

Gene expression datasets GSE182607 and GSE33327 were obtained from the GEO (https://www.ncbi.nlm.nih.gov/geo/) database. Differential expression analysis was conducted using GEO2R for GSE182607 (m6A-IP versus input) and GSE33327 (H19 high expression versus H19 low expression). Differentially expressed genes (DEGs) were identified using the criteria |log₂(fold-change)| ≥ 1 and *P* < 0.05. Functional annotation and pathway enrichment analyses were performed while employing the "clusterProfiler" package in R. Furthermore, the expression association between H19 and m6A reading proteins was determined using the Gene Expression Profiling Interactive Analysis (GEPIA, http://gepia.cancer-pku.cn/index. html) on the TCGA-CHOL dataset.

### Statistical analysis

GraphPad Prism 9 was used to analyze the data, and the Student's t-test was used to evaluate the differences between the two groups. The Chi-square (*χ²*) test was used to assess correlations between clinicopathological features and METTL3 expression in ICCA tissues. Pearson correlation analysis was performed to examine relationships between gene expression levels. The Kaplan-Meier method was employed to analyze overall survival, and the log-rank test was used to assess variations in survival curves. A *P* value < 0.05 was considered statistically significant in all analyses.

## Results

### Downregulation of METTL3 in ICCA is associated with adverse clinical outcomes

**Figure 1:**
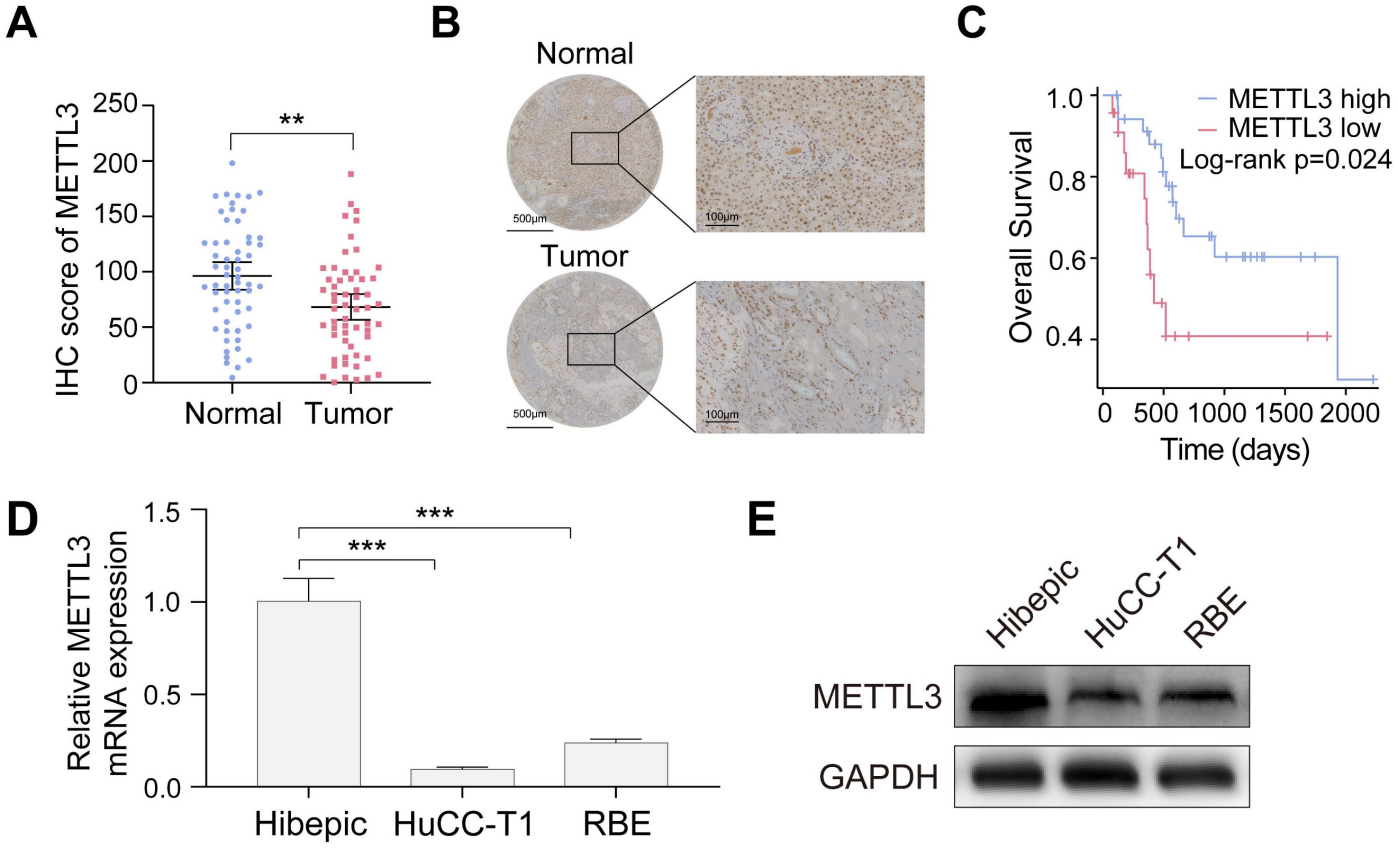
** METTL3 is down-expressed in ICCA and low expression of METTL3 is associated with poor prognosis. A.** METTL3 expression in the TJ cohort with 58 paired ICCA samples was analyzed by IHC staining. **B.** Representative IHC images of METTL3 in paired ICCA samples. **C.** Kaplan-Meier survival analysis of the correlation between METTL3 expression and overall survival in the TJ cohort. **D and E.** Endogenous METTL3 mRNA (D) and protein (E) expression level in the indicated cells were determined by RT-qPCR and western blot analysis, respectively. The results are presented as mean ± SD of three independent experiments. **P < 0.01, ***P < 0.001, according to a Student's t test.

To assess METTL3 expression in ICCA and its clinical relevance, 58 tumor samples and matched adjacent noncancerous tissues from the TJ cohort were analyzed. METTL3 expression was significantly lower in ICCA tissues than in normal tissues, according to the IHC assay (Figure 1A, B). Kaplan-Meier survival analysis demonstrated that patients with low METTL3 expression had significantly shorter OS (Figure 1C). Chi-square analysis showed that METTL3 expression was significantly associated with tumor size and hepatitis B surface antigen (HBsAg) status, but not with gender, age, hepatitis C core antigen (HCVAg), or tumor-node-metastasis (TNM) stage (Table 1). Moreover, RT-qPCR and western blot analyses confirmed that METTL3 expression was significantly lower in ICCA cell lines HuCC-T1 and RBE compared with human intrahepatic biliary epithelial (Hibepic) cells (Figure 1D, E). The results obtained suggest that METTL3 is downregulated in ICCA, and a poor prognosis is associated with its low expression. Further research with larger cohorts is required to evaluate METTL3 expression and their correlation with patient survival in ICCA samples.

### METTL3 inhibits the proliferation and migration of ICCA cells

**Figure 2:**
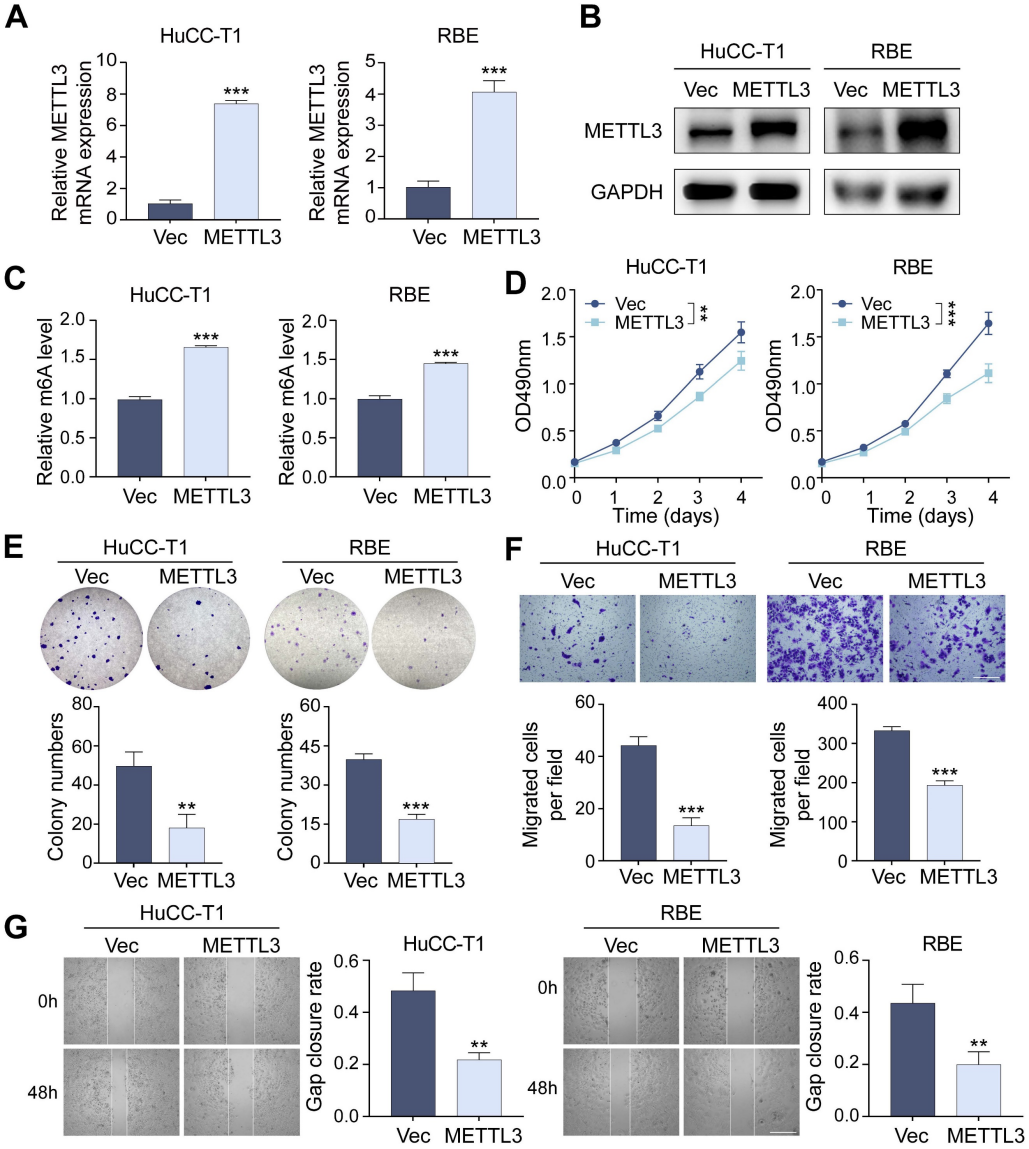
** Overexpression of METTL3 inhibits the proliferation and migration of ICCA cells. A and B.** The overexpression of METTL3 mRNA (A) and protein (B) level in HuCC-T1 and RBE cells was confirmed by RT-qPCR and western blot analysis, respectively. **C.** The total m6A level in METTL3-overexpressed HuCC-T1 and RBE cells was measured by m6A RNA methylation quantification assay. **D and E.** The proliferation of the indicated cells with METTL3 overexpression was measured by MTT (D) and colony formation (E) assay, respectively. **F and G.** The migration of the indicated cells with METTL3 overexpression was measured by transwell (F) and wound healing (G) assay, respectively. The results are presented as mean ± SD of three independent experiments. **P < 0.01, ***P < 0.001, according to a Student's t test. Scale bar, 100 μm.

Through lentivirus-mediated transfection, we produced stable METTL3-overexpressing HuCC-T1 and RBE cell lines to investigate the function of METTL3 in ICCA cells. The successful overexpression of METTL3 has been confirmed using RT-qPCR and western blot analyses (Figure 2A, B). We found that METTL3 overexpression significantly raised global m6A levels using a m6A RNA methylation quantification test, suggesting that METTL3's m6A catalytic activity remained intact (Figure 2C). MTT and colony formation assays revealed that METTL3 overexpression substantially inhibited ICCA cell proliferation (Figure 2D, E). Transwell migration and scratch-wound healing assays further demonstrated that METTL3 overexpression significantly inhibited ICCA cell migration (Figures 2F, G). At the molecular level, METTL3 overexpression increased E-cadherin expression while reducing N-cadherin and vimentin levels Figures S1A, B). In addition, the expression of metastasis-related markers matrix metallopeptidase 2 (MMP2), MMP9, and intercellular cell adhesion molecule-1 (ICAM1) was decreased, suggesting that METTL3 overexpression suppressed transcription of key tumor metastasis genes (Figure S1C).

**Figure 3:**
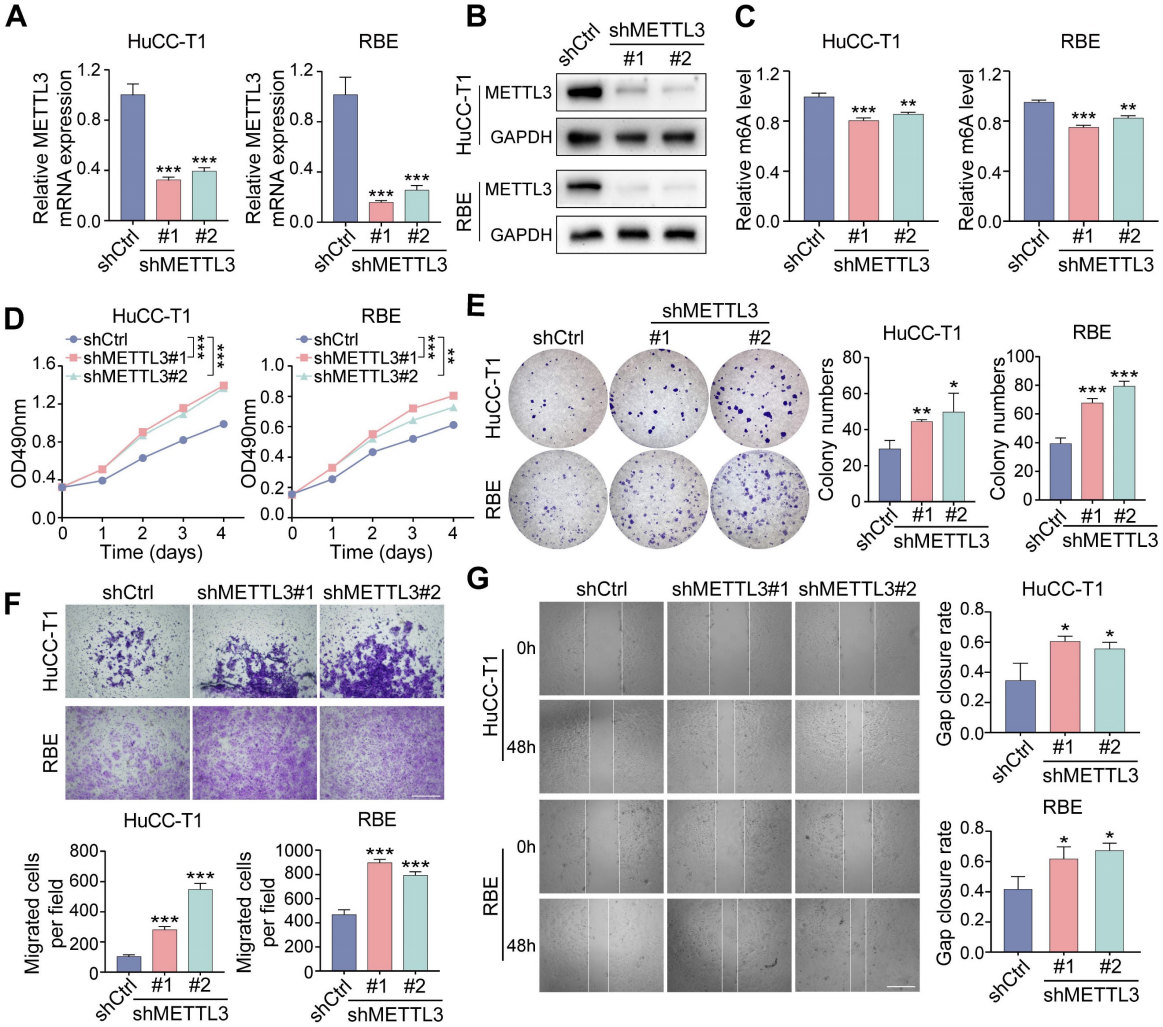
** Downregulation of METTL3 promotes the proliferation and migration of ICCA cells. A and B.** The downregulated METTL3 mRNA (A) and protein (B) level in HuCC-T1 and RBE cells was confirmed by RT-qPCR and western blot analysis, respectively. **C.** The total m6A level in METTL3-knockdown HuCC-T1 and RBE cells was measured by m6A RNA methylation quantification assay. **D and E.** The proliferation of the indicated cells with METTL3 knockdown was measured by MTT (D) and colony formation (E) assay, respectively. **F and G.** The migration of the indicated cells with METTL3 knockdown was measured by transwell (F) and wound healing (G) assay, respectively. The results are presented as mean ± SD of three independent experiments. *P < 0.05, **P < 0.01, ***P < 0.001, according to a Student's t test. Scale bar, 100 μm.

On the other hand, stable METTL3-knockdown HuCC-T1 and RBE cells were established using two independent shRNAs delivered via lentivirus (Figure 3A-C). METTL3 downregulation significantly enhanced cell proliferation (Figure 3D, E), increased migration in transwell assays (Figure 3F), and accelerated scratch-wound closure (Figure 3G). METTL3 knockdown decreased E-cadherin expression and increased N-cadherin and vimentin levels (Figure S1D, E), along with upregulation of MMP2, MMP9, and ICAM1 (Figure S1F). Analysis of gene expression datasets further confirmed a positive correlation between METTL3 and E-cadherin, as well as negative correlations with MMP2 and ICAM1 (Figure S1G).

These findings demonstrate that overexpression of METTL3 suppresses ICCA cell proliferation and migration, while METTL3 knockdown enhances these abilities.

### METTL3 regulates ICCA progression via its downstream target H19

**Figure 4:**
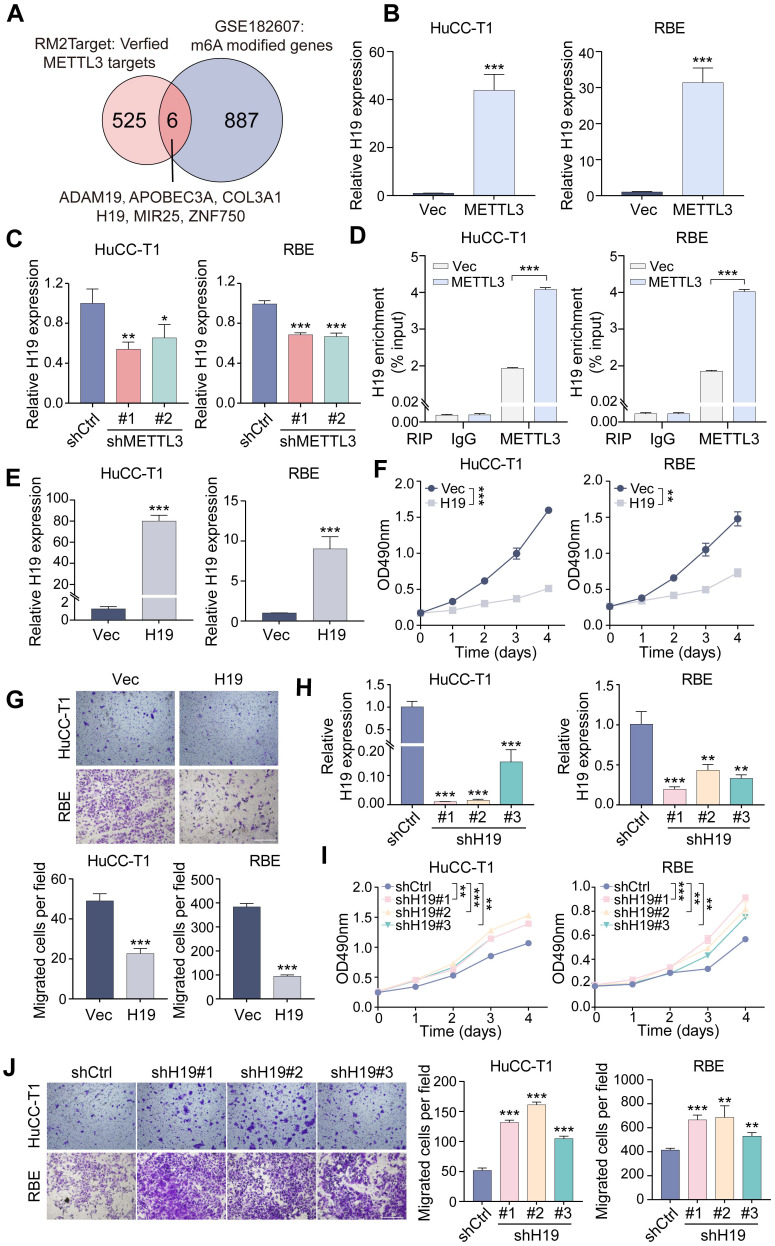
** H19 is the downstream target of METTL3 and functions on ICCA cell proliferation and migration. A.** Venn diagram showed 6 potential downstream genes of METTL3 using RM2Target and GSE182607 database analysis overlapping. **B and C.** The expression of H19 after METTL3 overexpression (B) or knockdown (C) in HuCC-T1 and RBE cells was examined by RT-qPCR. **D.** The interaction between METTL3 and H19 was evaluated by RIP-qPCR. **E.** The overexpression of H19 in HuCC-T1 and RBE cells was confirmed by RT-qPCR. **F.** The proliferation of the indicated cells with H19 overexpression was measured by MTT assay. **G.** The migration of the indicated cells with H19 overexpression was measured by transwell assay. **H.** The downregulated expression of H19 in HuCC-T1 and RBE cells was confirmed by RT-qPCR. **I.** The proliferation of the indicated cells with H19 knockdown was measured by MTT assay. **J.** The migration of the indicated cells with H19 knockdown was measured by transwell assay. The results are presented as mean ± SD of three independent experiments. *P < 0.05, **P < 0.01, ***P < 0.001, according to a Student's t test. Scale bar, 100 μm.

To identify potential downstream effectors of METTL3, we first retrieved 525 experimentally validated METTL3 targets from RM2Target (http://rm2target. canceromics.org/#/home). At the same time, 887 genes modified by m6A were identified through GEO2R analysis of dataset GSE182607, which contains MeRIP-seq results from METTL3-knockdown 293T cells. A Venn diagram was generated to identify overlapping genes between these two datasets (Figure 4A), highlighting the lncRNA H19 as a candidate, consistent with prior reports showing that METTL3 regulates H19 under hypoxic preconditioning (22. We then investigated whether METTL3 modulates H19 expression in ICCA cells. RT-qPCR analysis demonstrated that METTL3 overexpression upregulated H19 levels in HuCC-T1 and RBE cells, whereas METTL3 knockdown reduced H19 expression (Figure 4B, C). RIP-qPCR assay, which demonstrated elevated H19 enrichment upon METTL3 overexpression, further validated the direct interaction between METTL3 and H19 (Figure 4D).

Following, we investigated the functional role of H19 in ICCA. Stable H19-overexpressing HuCC-T1 and RBE cell lines were generated via lentivirus-mediated transfection (Figure 4E). H19 overexpression significantly suppressed ICCA cell proliferation and migration (Figure 4F, G and Figure S2A, B). To further validate these phenotypic effects, three shRNAs targeting H19 were designed and used to reduce H19 expression in HuCC-T1 and RBE cells via lentiviral infection (Figure 4H). In comparison to H19-overexpressing cells, H19 knockdown enhanced proliferation and migration compared with control cells (Figure 4I, J and Figure S2C, D). H19 downregulation had the opposite impact from H19 overexpression, which enhanced E-cadherin and lowered N-cadherin expression Figure S2E).

**Figure 5:**
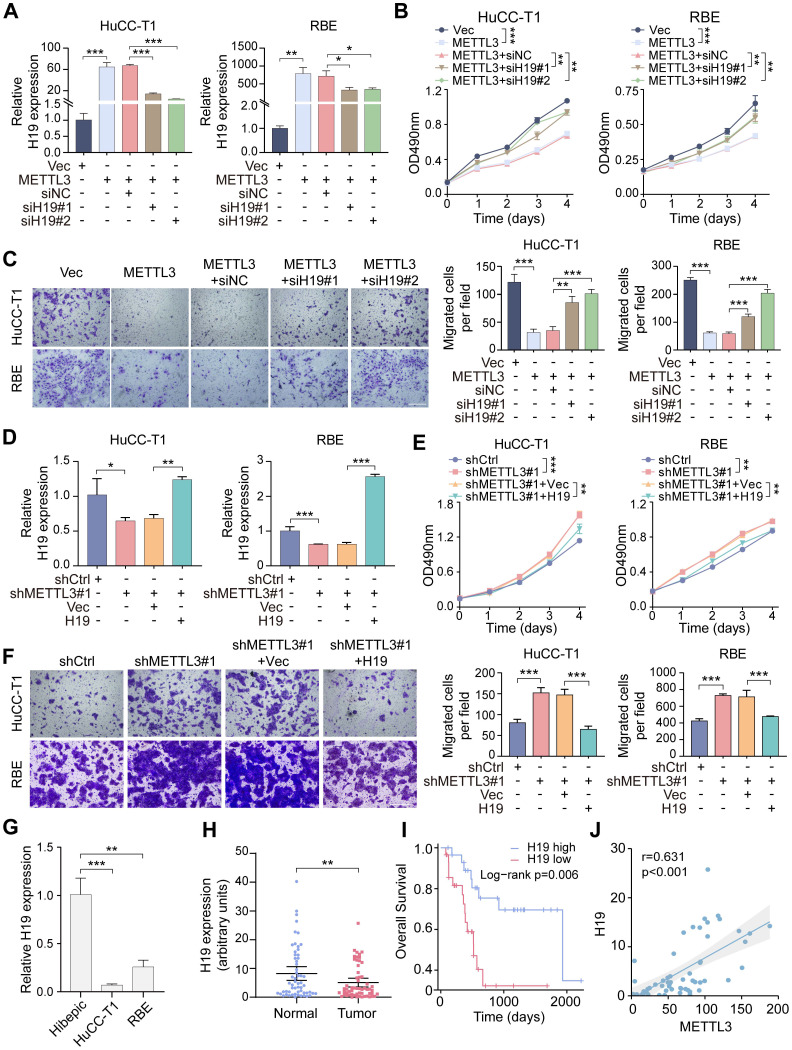
** H19 mediates the impacts of METTL3 in ICCA cells. A.** The downregulation of H19 expression by siRNAs in the METTL3-overexpressed HuCC-T1 and RBE cells was confirmed by RT-qPCR. **B.** The proliferation of the indicated cells with METTL3 overexpression and H19 silencing was measured by MTT assay. **C.** The migration of the indicated cells with METTL3 overexpression and H19 silencing was measured by transwell assay. **D.** The overexpression of H19 in the METTL3-knockdown HuCC-T1 and RBE cells was confirmed by RT-qPCR. **E.** The proliferation of the indicated cells with METTL3 knockdown and H19 overexpression was measured by MTT assay. **F.** The migration of the indicated cells with METTL3 knockdown and H19 overexpression was measured by transwell assay. **G.** Endogenous H19 expression in the indicated cells was determined by RT-qPCR. **H.** H19 expression in the paired ICCA samples was examined by RT-qPCR. **I.** Kaplan-Meier survival analysis of the correlation between H19 expression and overall survival in the TJ cohort. **J.** The correlation between METTL3 and H19 expression in the TJ cohort. The results are presented as mean ± SD of three independent experiments. *P < 0.05, **P < 0.01, ***P < 0.001, according to a Student's t test. Scale bar, 100 μm.

To determine whether H19 mediates METTL3-driven effects in ICCA, H19 was silenced using two specific siRNAs in METTL3-overexpressing cells (Figure 5A). MTT and transwell assays demonstrated that H19 knockdown rescued the inhibitory effects of METTL3 overexpression on cell proliferation and migration (Figure 5B, C). On the other hand, H19 overexpression in METTL3-knockdown cells reversed the enhanced proliferation and migration induced by METTL3 downregulation (Figure 5D-F). These results suggest that H19 serves as a crucial downstream mediator of METTL3 in regulating ICCA cell proliferation and migration.

Furthermore, ICCA cell lines RBE and HuCC-T1 demonstrated significantly reduced levels of H19 expression in comparison to Hibepic cells (Figure 5G). To assess the clinical relevance of H19 in ICCA, RT-qPCR analysis was performed on paired tumor and adjacent normal tissues from the TJ cohort. Compared to normal tissues, tumor tissues had considerably lower H19 levels (Figure 5H). According to Kaplan-Meier survival analysis, patients with low levels of H19 expression had shorter OS (Figure 5I). Importantly, H19 expression showed a positive correlation with METTL3 levels (Figure 5J).

### METTL3 promotes the expression of H19 in an m6A-dependent manner, and IGF2BP1 enhances H19 stability

We then explored the mechanism by which METTL3 modulates H19 expression. Using the SRAMP online tool, potential m6A modification sites within H19 were predicted (Supplementary Table 4.

**Figure 6:**
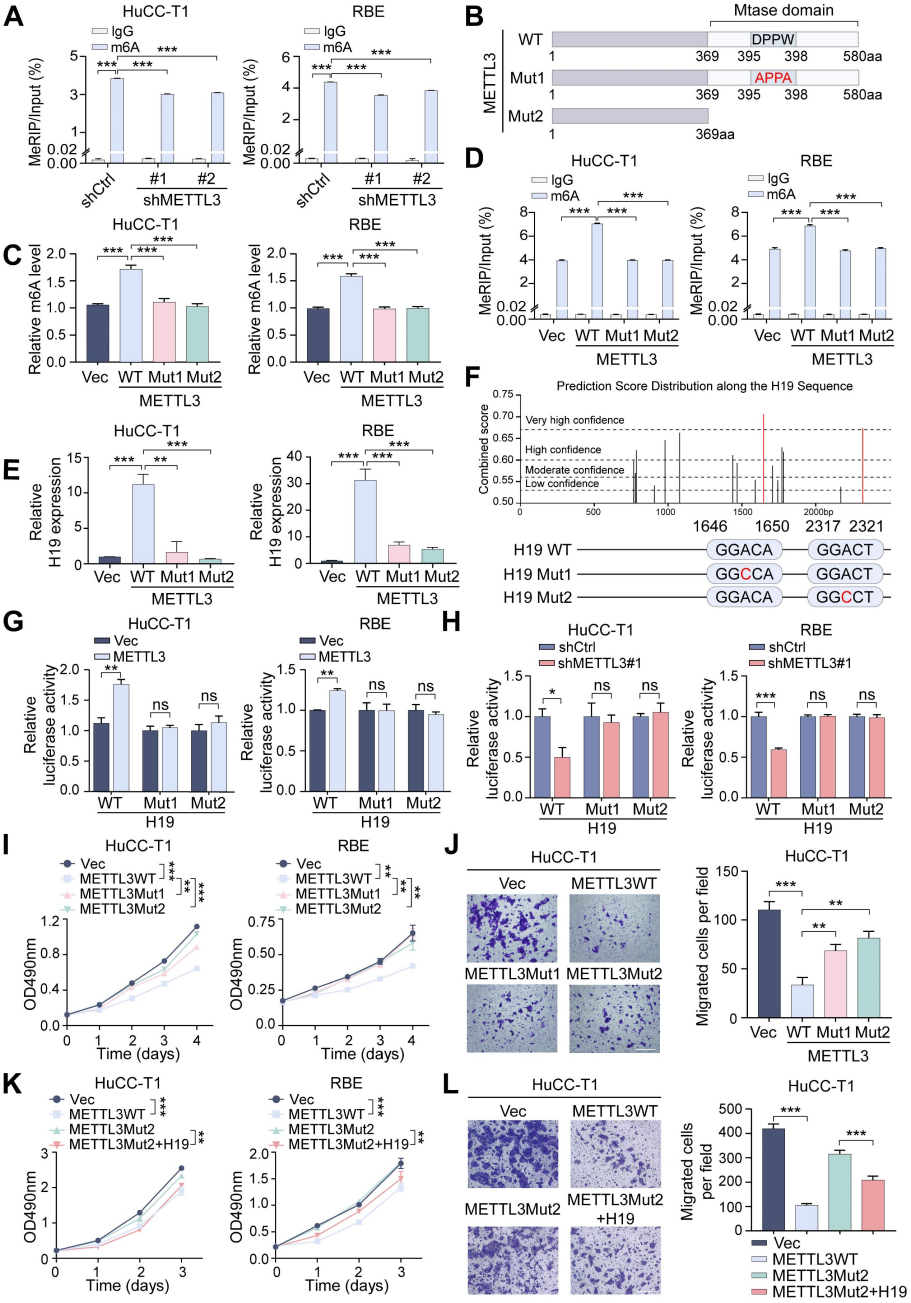
** METTL3 regulates the expression of H19 in an m6A-mediated manner. A.** MeRIP-qPCR analysis was applied to assess the m6A modification of H19 in METTL3-knockdown HuCC-T1 and RBE cells. **B.** Mutation or deletion of the methyltransferase domain (Mtase) in the METTL3 coding sequence. **C.** The total m6A level in METTL3 wild-type- or mutant-overexpressed cells was measured by m6A RNA methylation quantification assay. **D.** The m6A modification of H19 was detected by MeRIP-qPCR in the HuCC-T1 and RBE cells with METTL3 mutants overexpression. **E.** The expression of H19 in METTL3 wild-type- or mutant-overexpressed cells was evaluated by RT-qPCR. **F.** Mutation of the predicated m6A modification sites in the H19 sequence. **G and H.** Relative activity of the H19 wild-type- or mutant-luciferase reporters in METTL3-overexpressed (G) or METTL3-knockdown (H) HuCC-T1 and RBE cells was determined. **I.** The proliferation of the indicated cells with METTL3 mutants overexpression was measured by MTT assay. **J.** The migration of HuCC-T1 cells with METTL3 mutants overexpression was measured by transwell assay. **K.** The proliferation of the indicated cells with METTL3 mutants overexpression and H19 overexpression was measured by MTT assay. **L.** The migration of HuCC-T1 cells with METTL3 mutants overexpression and H19 overexpression was measured by transwell assay. The results are presented as mean ± SD of three independent experiments. *P < 0.05, **P < 0.01, ***P < 0.001, ns, no significance, according to a Student's t test. Scale bar, 100 μm.

To determine whether METTL3 mediates H19 m6A modification, MeRIP-qPCR was performed. The m6A-specific antibody greatly enriched H19 in comparison to the IgG control, and after METTL3 knockdown, the m6A-modified level of H19 was much lower (Figure 6A). To assess the role of METTL3's catalytic activity, two mutant METTL3 plasmids were constructed: Mut1 (aa395-398, DPPW → APPA) and Mut2 (deletion of the methyltransferase domain) (Figure 6B). In HuCC-T1 and RBE cells, overexpression of these catalytic mutants resulted in a dramatic reduction of total m6A levels compared with wild-type METTL3-overexpressing cells (Figure 6C). MeRIP-qPCR showed that mutant METTL3 failed to increase the m6A modification of H19 (Figure 6D), and RT-qPCR confirmed that H19 expression was not elevated by mutant METTL3 (Figure 6E). The results obtained suggest that METTL3 regulates H19 expression in a manner dependent on m6A.

Prediction analysis identified a total of 16 potential m6A modification sites within the H19 sequence, with the sites at 1648 bp and 2319 bp showing the highest confidence scores Supplementary Table 4. Using luciferase reporter plasmids that included either the wild-type H19 sequence or two mutant versions, we were able to examine the functional importance of these m6A sites in controlling H19 expression. The wild-type construct retained all m6A sites, whereas in the mutant constructs, the adenosine residues at positions 1648 bp and 2319 bp were replaced with cytosine (Figure 6F). Dual-luciferase reporter assays revealed that METTL3 overexpression significantly increased relative luciferase activity in wild-type H19-transfected cells, while METTL3 knockdown reduced it. In comparison, luciferase activity in cells transfected with the mutant H19 constructs was unresponsive to METTL3 modulation (Figure 6G, H). These findings indicate that the 1648 bp and 2319 bp sites are functional m6A modification sites of H19 that METTL3 directly regulates.

The above results indicate that METTL3's m6A catalytic activity is crucial for regulating H19. To further determine whether this catalytic function is necessary for METTL3's physiological role in ICCA, we examined the effects of mutant METTL3 on cell behavior. Compared with wild-type METTL3, the catalytic mutants exhibited a markedly reduced ability to suppress ICCA cell proliferation and migration (Figure 6I, J and Figure S3A). Notably, overexpression of H19 rescued the impaired inhibitory effects of mutant METTL3, restoring suppression of cell proliferation and migration (Figure 6K, L and Figure S3B). These results demonstrate that METTL3 regulates H19 to carry out its tumor-suppressive action in ICCA in an m6A-dependent way.

Given that m6A modification often influences RNA stability and thereby regulates transcript levels, we next examined whether METTL3-mediated m6A affects H19 stability. Actinomycin D, a transcription inhibitor, was administered to ICCA cells for the specified durations. H19 stability was significantly enhanced following METTL3 overexpression, whereas METTL3 knockdown led to accelerated H19 decay compared with controls Figure S3C, D). We then explored which m6A reader proteins mediate this effect. siRNAs were used to individually knock down members of the YTHDF (YTHDF1/2/3) and IGF2BP (IGF2BP1/2/3) families (Figure S4A), followed by actinomycin D treatment. Knockdown of IGF2BP1 but not YTHDF1/2/3 or IGF2BP2/3 significantly accelerated H19 degradation (Figure S4B). RIP assays confirmed that IGF2BP1 directly bound to H19 (Figure S4C). Correlation analysis revealed a strong positive relationship between IGF2BP1 and H19 expression, whereas other readers showed no significant correlation (Figure S4D).

To further validate IGF2BP1 as the m6A reader of H19, IGF2BP1 was silenced, resulting in decreased H19 expression (Figure S4E, F) and reduced H19 enrichment in RIP pull-downs (Figure S4G). Moreover, H19 decay was significantly accelerated after IGF2BP1 knockdown, an effect that was partially rescued by METTL3 overexpression (Figure S4H). It can be inferred from these results that IGF2BP1 serves as a crucial m6A reader in the METTL3-H19 regulatory axis, thereby stabilizing H19 transcripts in ICCA cells.

### H19 affects ICCA progression through regulating the expression of PPARγ

**Figure 7:**
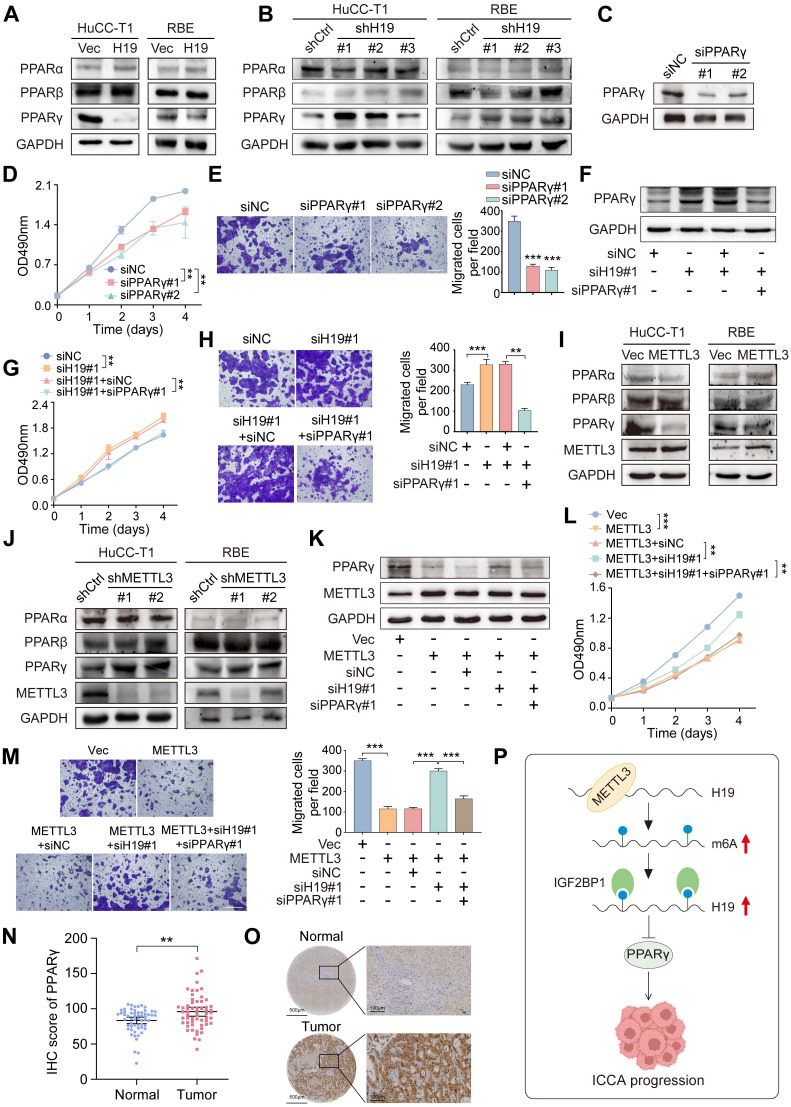
** METTL3 affects ICCA progression through the H19 and PPARγ signaling. A and B.** The protein expression of PPARα, PPARβ and PPARγ was determined by western blot in HuCC-T1 and RBE cells with H19 overexpression (A) or knockdown (B). **C.** PPARγ protein expression was determined by western blot in HuCC-T1 cells with PPARγ siRNAs transfection. **D and E.** The proliferation and migration of HuCC-T1 cells with PPARγ silencing was detected by MTT (D) and transwell (E) assay, respectively. **F.** PPARγ protein expression was determined by western blot in HuCC-T1 cells with the indicated siRNAs treatment. **G and H.** The proliferation and migration of HuCC-T1 cells with the indicated siRNAs treatment was detected by MTT (G) and transwell (H) assay, respectively. **I and J.** The protein expression of PPARα, PPARβ and PPARγ was determined by western blot in HuCC-T1 and RBE cells with METTL3 overexpression (I) or knockdown (J). **K.** PPARγ protein expression was determined by western blot in HuCC-T1 cells with METTL3 overexpression and the indicated siRNAs treatment. **L and M.** The proliferation and migration of HuCC-T1 cells with METTL3 overexpression and the indicated siRNAs treatment was detected by MTT (L) and transwell (M) assay, respectively. **N.** PPARγ expression in the 58 paired ICCA samples was analysed by IHC staining. **O.** Representative IHC images of PPARγ in paired ICCA samples. **P.** Schematic model according to the results of this study. The results are presented as mean ± SD of three independent experiments. **P < 0.01, ***P < 0.001, according to a Student's t test. Scale bar, 100 μm.

A total of 32 differentially expressed genes linked to H19 were found after the GSE33327 dataset's H19 expression levels were divided into high and low groups. KEGG pathway analysis revealed enrichment in several pathways, including the PPAR signaling pathway (Figure S5A). Previous studies have shown that liver malignancies are often accompanied by dysregulated lipid and glucose metabolism, with the PPAR signaling pathway playing a central role in cellular metabolic regulation (23,24. Based on this, we hypothesized that H19 may influence ICCA progression by modulating the PPAR pathway. Among the three main PPAR subtypes, PPARα, PPARβ, and PPARγ 25, we observed that H19 overexpression in HuCC-T1 and RBE cells selectively decreased PPARγ levels, whereas H19 knockdown upregulated PPARγ, with no significant changes in PPARα or PPARβ expression (Figures 7A, B and Figure S5B, C). Functional assays demonstrated that PPARγ downregulation inhibited ICCA cell proliferation and migration (Figure 7C-E and Figure S5D). Furthermore, simultaneous knockdown of H19 and PPARγ in HuCC-T1 cells partially reversed the enhanced proliferation and migration induced by H19 disruption (Figure 7F-H and Figure S5E). This suggests that H19 modulates ICCA cell progression, at least in part, by regulating PPARγ.

Overexpression of METTL3 led to downregulation of PPARγ, whereas METTL3 knockdown resulted in increased PPARγ levels (Figure 7I, J and Figure S5F, G). Functionally, METTL3 overexpression significantly suppressed ICCA cell proliferation and migration, effects that were reversed upon H19 knockdown. Subsequent knockdown of PPARγ restored the inhibitory effect on cell proliferation and migration (Figure 7K-M and Figure S5H), indicating that ICCA progression is regulated via the METTL3-H19-PPARγ axis. METTL3 and PPARγ showed a strong negative association, but PPARα and PPARβ showed no discernible link, according to an analysis of gene expression datasets Figure S5I). Consistently, IHC analysis of the TJ cohort demonstrated that PPARγ expression was markedly elevated in tumors, and higher PPARγ levels were associated with poorer OS (Figure 7N, O and Figure S5J). Moreover, both H19 and METTL3 expression levels negatively correlated with PPARγ expression (Figure S5K, L).

Collectively, these findings demonstrate that METTL3 promotes H19 stability and expression through an m6A-IGF2BP1-dependent pathway, and that the METTL3-H19 axis subsequently reduces PPARγ, ultimately decreasing ICCA cell proliferation and migration (Figure 7P).

### Oncolytic adenovirus-mediated H19 overexpression inhibits ICCA growth

**Figure 8:**
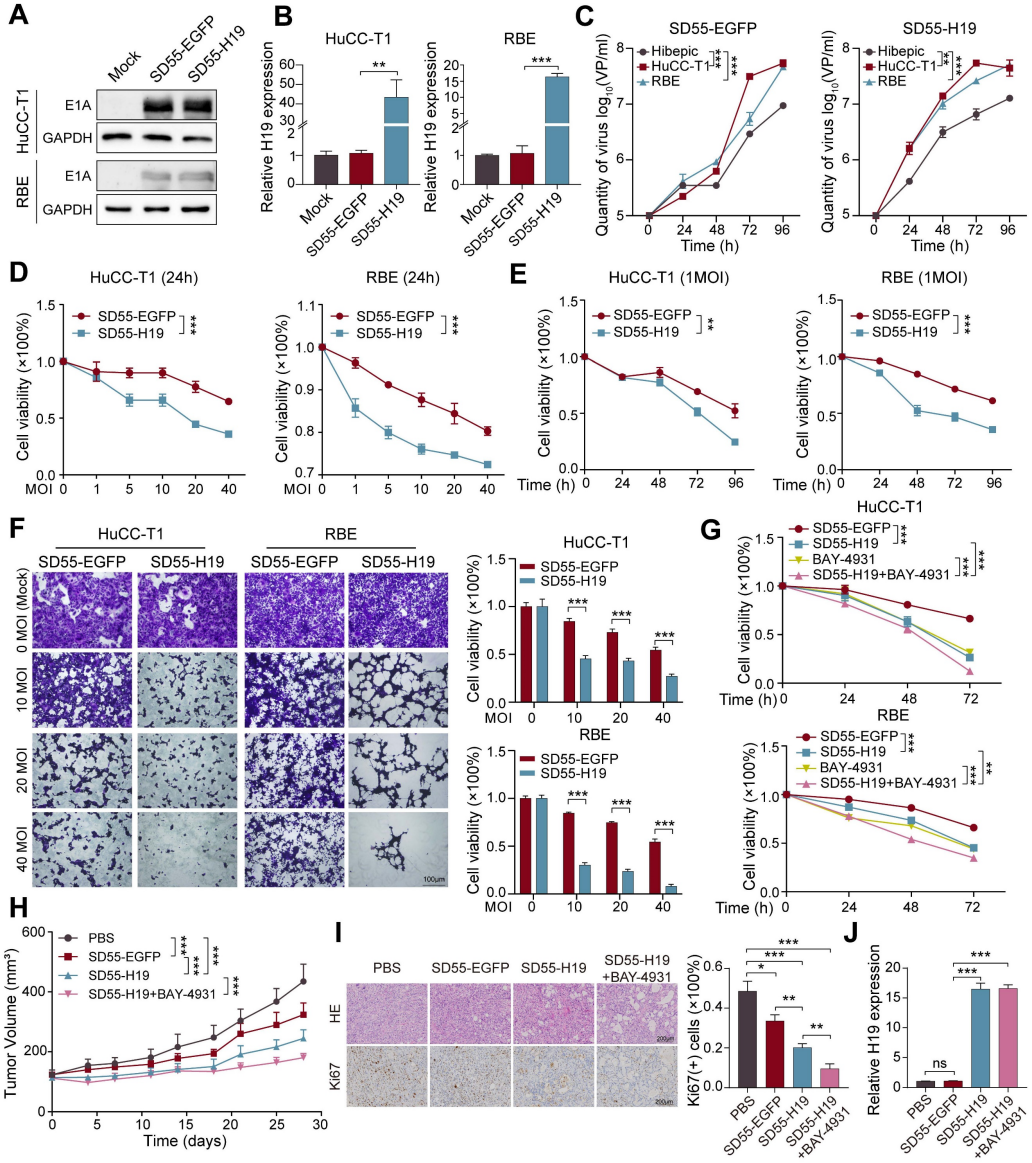
** Oncolytic adenovirus-mediated H19 overexpression inhibits ICCA cell growth. A.** Expression of adenoviral E1A protein in SD55-EGFP- or SD55-H19-infected HuCC-T1 and RBE cells was detected by western blot. **B.** Expression of H19 in SD55-EGFP- or SD55-H19-infected HuCC-T1 and RBE cells was detected by RT-qPCR. **C.** HuCC-T1, RBE and Hibepic cells were infected with SD55-EGFP or SD55-H19 (1MOI). Viral titer was measured by viral progeny assay at the indicated infection time. **D and E.** HuCC-T1 and RBE cells were infected with SD55-EGFP or SD55-H19 at the indicated MOI (D) or at the indicated infection time (E), and MTT assay was performed to detect cell viability. Cell viability was shown as OD490nm value fold change relative to that of mock cells. **F.** The cytopathic effect was detected by crystal violet staining in HuCC-T1 and RBE cells infected with SD55-EGFP or SD55-H19 at the indicated MOI for 48 hours with representative images shown. The stained cell numbers were counted, and cell viability was defined as fold change relative to that of mock cells. **G.** The cytotoxicity of SD55-H19 (1MOI) combined with BAY-4931 (2μM) on HuCC-T1 and RBE cells was detected by MTT assay. **H.** Tumor volume of HuCC-T1 xenografts (n=8). **I.** Representative HE staining and immunohistochemistry staining images of Ki67 in the indicated tumor tissues, and quantification of Ki67 positive cells. **J.** RT-qPCR detection of H19 expression in tumor tissues from treated mice. The results are presented as mean ± SD of three independent experiments. *P < 0.05, **P < 0.01, ***P < 0.001, ns, no significance, according to a Student's t test.

One promising anticancer tactic is the use of oncolytic adenoviruses, which reproduce and lyse specifically in tumor cells (26. To explore the therapeutic potential of H19 in ICCA, we employed an oncolytic adenovirus to deliver H19. The H19 expression cassette was inserted into the viral E1B55-kDa deletion region, which enhances tumor-selective cytotoxicity 27, generating the recombinant virus SD55-H19 (with SD55-EGFP serving as a control; Figure S6A). To confirm virus functionality, E1A protein expression was assessed in ICCA cells infected with SD55-H19 or SD55-EGFP via western blot, demonstrating robust E1A expression and efficient viral replication in ICCA cells (Figure 8A). RT-qPCR further validated H19 overexpression mediated by the virus (Figure 8B). Tumor-specific replication of the oncolytic adenoviruses was then evaluated, revealing significantly higher replication of SD55-H19 and SD55-EGFP in ICCA tumor cells compared with normal Hibepic cells, indicating effective tumor-targeting capacity of the recombinant viruses (Figure 8C).

To assess the *in vitro* tumoricidal effect of the recombinant viruses, RBE and HuCC-T1 cells were infected with SD55-H19 or SD55-EGFP for 24 h at the indicated MOIs. MTT assays demonstrated that SD55-H19 induced dose-dependent cytotoxicity, which was significantly greater than that of SD55-EGFP (Figure 8D). At an MOI of 1, SD55-H19 inhibited ICCA cell growth in a time-dependent manner compared with SD55-EGFP (Figure 8E). Cytopathic effects were further evaluated by staining adherent live cells with crystal violet, revealing that SD55-H19 elicited a more pronounced cytopathic effect on ICCA cells than SD55-EGFP (Figure 8F). Given that H19 exerts its effect by inhibiting PPARγ, and that PPARγ inhibition suppresses cell proliferation, we combined the PPARγ inhibitor BAY-4931 28 with SD55-H19. The combination treatment exhibited stronger cytotoxicity than either SD55-H19 or BAY-4931 alone (Figure 8G and Figure S6B). Collectively, these results indicate that SD55-H19 is more effective than SD55-EGFP in eliminating ICCA cells *in vitro*, and that its combination with BAY-4931 further enhances antitumor efficacy.

The antitumor efficacy of the recombinant viruses was further evaluated *in vivo* using HuCC-T1 tumor xenografts in mice Figure S6C). SD55-H19 demonstrated stronger tumor growth inhibition than the control SD55-EGFP virus, and the combination of SD55-H19 with BAY-4931 significantly suppressed tumor xenograft growth compared with SD55-H19 alone (Figure 8H). Histopathological analysis using HE staining revealed that the combination treatment induced the most extensive tumor cell death and necrotic changes, followed by SD55-H19 and SD55-EGFP treatments (Figure 8I). IHC staining showed that the proliferation marker Ki67 was significantly reduced in tumors treated with SD55-H19 plus BAY-4931 compared with either SD55-H19 or SD55-EGFP alone (Figure 8I). RT-qPCR analysis confirmed efficient overexpression of H19 in the tumor tissues (Figure 8J). These findings suggest that the H19-armed oncolytic adenovirus, particularly when combined with PPARγ inhibition, enhances antitumor efficacy against ICCA *in vivo*.

## Discussion

The N6-methyladenosine (m6A) is the most prevalent chemical modification on mRNA and non-coding RNA. Emerging evidence has demonstrated that m6A changes can influence the initiation and progression of various cancers, including pancreatic (29, lung 30, colorectal 31, and breast 32 cancers, by modulating the expression of oncogenes or tumor suppressor genes. The m6A process is orchestrated by "writers," "erasers," and "readers," among which METTL3, a core component of the methyltransferase complex, plays a crucial regulatory role. We observed that METTL3 expression was downregulated in ICCA, and this reduction was significantly associated with enhanced tumor proliferation and migration. Mechanistically, METTL3 was found to regulate H19 through an m6A-IGF2BP1-dependent pathway positively. The METTL3-H19 axis subsequently modulated ICCA cell proliferation and migration by suppressing PPARγ signaling. Importantly, our findings highlight H19 as a promising therapeutic target for ICCA.

Among the m6A modulators, METTL3 has been extensively studied. Previous studies have reported a tumor-suppressive role of METTL3 in glioblastoma stem cells, endometrial cancer, and ocular melanoma 33-35. On the other hand, several independent investigations have revealed an oncogenic function of METTL3 in breast, colorectal, and lung cancers 36-38. Wei et al. demonstrated that METTL3 ablation in established liver tumors or hepatoma cells reduced tumor growth; however, deletion of METTL3 in the mouse liver promoted carcinogenesis in both oncogene-driven and chemical carcinogen-induced models 39. These seemingly contradictory findings indicate that METTL3 exerts context-dependent effects, influenced by tissue type, cell lineage, and spatiotemporal factors. In our study, METTL3 was downregulated in ICCA, and its low expression correlated with poor patient prognosis. Furthermore, functional experiments demonstrated that METTL3 overexpression inhibited the migration and proliferation of ICCA cells. Collectively, these outcomes support a tumor-suppressive role for METTL3 in ICCA and underscore the broader impact of METTL3-mediated m6A methylation on ICCA growth.

The H19 is a long non-coding RNA implicated in a wide range of cancers, including breast 40, hepatocellular carcinoma 41, lung 42, and neuroendocrine prostate cancer 43, as well as other metabolic diseases, such as diabetes mellitus 44. Furthermore, H19 influences angiogenesis, apoptosis, cell proliferation, and metastasis, among other aspects of tumor biology. Interestingly, H19 has also been reported to exert tumor-suppressive effects in several cancer types, including liver cancer 45, retinoblastoma 46, and prostate cancer 47. H19 was identified as a downstream target of METTL3 in ICCA in the current investigation. In line with earlier research by Yang et al., which demonstrated that METTL3 increased the m6A modification of H19, thereby promoting inflammation and pyroptosis in atherosclerosis, METTL3 enhanced H19 expression 48. Using catalytically inactive METTL3 mutants, we confirmed that METTL3 regulated H19 via m6A modification and further pinpointed two critical H19 m6A sites, located at 1648 bp and 2319 bp, responsible for this regulation. It is well established that m6A "reader" proteins recognize methylation sites to mediate RNA stability, with the YTHDF and IGF2BP families being major regulators of methylated RNA 49. The present data demonstrated that IGF2BP1, but not other readers, directly bound to H19 in ICCA cells. Importantly, IGF2BP1 has been reported to modulate tumor progression by stabilizing target lncRNAs in gastric cancer 50. Furthermore, it has been shown that IGF2BP1 stabilizes H19 and mediates the METTL3-dependent upregulation of H19 via the m6A-IGF2BP1 axis, providing a mechanistic explanation for the increased H19 expression following m6A modification.

The METTL3-H19 axis' downstream molecular mechanism was further examined in the current study, which also revealed that the PPAR signaling pathway exhibited a significant enrichment of H19-regulated genes. Members of the nuclear receptor superfamily, PPARs are essential for several physiological and pathological processes, including inflammation, cancer, and cell metabolism 51-53. PPARs are currently classified into three subtypes: PPARα, PPARβ, and PPARγ 54. Our data revealed that both METTL3 and H19 exerted strong negative regulatory effects specifically on PPARγ. Moreover, Liu et al. revealed that PPARγ-mediated lipid metabolic reprogramming promoted lymph node metastasis and ICCA progression 55. In line with this, we observed that PPARγ was abnormally upregulated in ICCA, and knockdown of H19 reversed the suppressive effects of METTL3 overexpression on PPARγ, thereby restoring the proliferative and migratory capacity of ICCA cells.

Oncolytic adenoviruses, which are double-stranded DNA viruses, have been widely applied in gene therapy 56,57. Considering the tumor-suppressive role of H19 in ICCA, we engineered an oncolytic adenovirus to overexpress H19. The resulting SD55-H19 virus exhibited selective targeting of ICCA cells and demonstrated potent tumoricidal activity. Furthermore, combining SD55-H19 with a PPARγ inhibitor enhanced the anti-proliferative effects in ICCA *in vitro* and *in vivo*. Despite the advantages of oncolytic adenoviruses, including high infection efficiency, a large capacity for exogenous genes, ease of large-scale production, and low pathogenicity 58, 59, further improvements to the SD55 vector could enhance its *in vivo* transduction efficiency. Such modifications may involve two strategies: 1 transcriptional targeting, by controlling the expression of the adenoviral replication-essential gene E1A under a liver-specific promoter; and 2 transductional targeting, by altering the adenoviral fiber with alternative serotypes such as type 11, or engineering the adenoviral capsid with specific ligands to enable selective binding to surface receptors on ICCA cells.

In summary, METTL3 expression is downregulated in ICCA, and it promotes the m6A modification of H19, thereby enhancing H19 expression in an IGF2BP1-dependent manner. H19, in turn, modulates PPARγ signaling to suppress ICCA progression. Furthermore, oncolytic adenovirus-mediated H19 overexpression, when combined with a PPARγ inhibitor, exhibits potent antitumor effects in ICCA xenograft models. The present results suggest a potential therapeutic approach for treating ICCA and highlight the crucial role that m6A regulators play in ICCA development.

## Supplementary Material

Supplementary figures and tables.

## Figures and Tables

**Table 1:** Relationship between METTL3 and clinicopathologic features of ICCA patients.

Variables		METTL3 expression		P value
		Low	High	
Gender	Male	16	18	0.4351
	Female	14	10	
Age (y)	≤60	12	10	0.7913
	>60	18	18	
HBsAg	Yes	22	12	**0.0320**
	No	8	16	
HCVAg	Yes	1	2	0.6053
	No	29	26	
Tumor size (cm)	≤5	9	18	**0.0170**
	>5	21	10	
TNM stage	I, II	20	19	1.0000
	III, IV	10	9	

## Abbreviations

ICCA: intrahepatic cholangiocarcinoma
M6A: N6-methyladenosine
PPARγ: peroxisome proliferator-activated receptor gamma
FGFR2: fibroblast growth factor receptor 2
IDH1: isocitrate dehydrogenase 1
METTL3: methyltransferase-like 3
METTL14: methyltransferase-like 14
WTAP: wilms tumor 1-associated protein
ALKBH5: alkylation repair homolog protein 5
FTO: fat mass and obesity-associated protein
YTHDF: YTH domain family proteins
IGF2BP: insulin-like growth factor 2 mRNA-binding proteins
LncRNA: long non-coding RNA
FFPE: formalin-fixed paraffin-embedded
OS: overall survival
ORF: open reading frame
shRNA: short hairpin RNA
RT-qPCR: real-time quantitative PCR
cDNA: complementary DNA
MTT: 3-[4,5-dimethylthiazol-2-yl]-2,5 diphenyl tetrazolium bromide
DMSO: dimethyl sulfoxide
RIP: RNA immunoprecipitation
MeRIP: methylated RNA immunoprecipitation
MOI: multiplicities of infection
HE: hematoxylin and eosin
IHC: immunohistochemistry
TMA: tissue microarray
HBsAg: hepatitis B surface antigen
HCVAg: hepatitis C core antigen
TNM: tumor-node-metastasis
MMP2: matrix metallopeptidase 2
ICAM1: intercellular cell adhesion molecule-1
PFU: plaque forming unit
