# Supplementary material for: METTL3-mediated m6A methylation on lncRNA H19 inhibits intrahepatic cholangiocarcinoma progression through PPARγ downregulation
Source: Int J Biol Sci. 2025 Sep 29;21(14):6062–80. doi: 10.7150/ijbs.120413 (PMC12594553; doi:10.7150/ijbs.120413)
Supplement: Supplementary file 1 — Supplementary figures and tables. [file ijbsv21p6062s1.pdf]

Supplemental information

METTL3-mediated m6A methylation on lncRNA H19 inhibits intrahepatic cholangiocarcinoma progression through PPARγ downregulation

Rong Xiao[1,2], Xinya Lu[1], Fang Huang[3], Yaru Zhao[1], Hao Jin[1], Xiaoyuan Jia[1], Biao Huang[1], Yigang Wang[*,1], Liang Chu[*,2]

1, College of Life Sciences and Medicine, Zhejiang Sci-Tech University, Hangzhou 310018, China; Oncology Department, Zhejiang Sci-Tech University Shaoxing Academy of Biomedicine, Shaoxing 312366, China

2, Hepatic Surgery Center, Tongji Hospital, Tongji Medical College, Huazhong University of Science and Technology, Wuhan 430030, China

3, Cancer Center, Department of Pathology, Zhejiang Provincial People's Hospital, Affiliated People's Hospital, Hangzhou Medical College, Hangzhou 310014, China

*Correspondence: Yigang Wang, College of Life Sciences and Medicine, Zhejiang Sci-Tech University, No. 928, No. 2 Street, Hangzhou, Zhejiang, 310018, China. Telephone: 86-571-86843187. E-mail: ygwang@zstu.edu.cn;

Liang Chu, Hepatic Surgery Center, Tongji Hospital, Tongji Medical College, Huazhong University of Science and Technology, No. 1095 Jiefang Avenue, Wuhan, Hubei, 430030, China. Telephone: 86-27-69378518. E-mail: liangchu@tjh.tjmu.edu.cn.

Supplementary Table 1. Key resources.

Reagent or resource	Source	Identifier
HuCC-T1	RIKEN BioResource Research Center	RCB1960
RBE	RIKEN BioResource Research Center	RCB1292
293T	Type Culture Collection of the Chinese Academy of Sciences	SCSP-502
239A	Type Culture Collection of the Chinese Academy of Sciences	SCSP-5094
Hibepic	Procell Life Science and Technology	CP-H042
RPMI 1640 medium	Gibco	C11875500BT
Fetal bovine serum	Excell	FCS500
DMEM medium	Gibco	C11995500BT
Lipofectamine 2000	Invitrogen	11668027
Lentivirus concentration solution	Beyotime	C2901M
Puromycin	MedChemExpress	HY-B1743
TRIzol reagent	Generay	GK3016
HiFiScript cDNA Synthesis kit	CWBIO	CW2569M
SYBR qPCR Master Mix	Vazyme	Q311-02
RIPA buffer	Beyotime	P0013B
BCA protein assay kit	Thermo	23227
0.45μm PVDF membranes	Millipore	IPVH08100
ECL imaging system	Vazyme	E411-04
Actinomycin D	MedChemExpress	HY-17559
Luc-Pair Duo-Luciferase Assay Kit	GeneCopoeia	LF003
Magna RIP kit	Millipore	17-700
EpiQuik m6A RNA Methylation Quantification Kit	Epigentek	P-9005-48
Magna MeRIP m6A Kit	Millipore	17-10499
BAY-4931	MedChemExpress	HY-148352
NOD/SCID	GemPharmatech	T001492

Supplementary Table 2. Sequences of siRNA, shRNA and RT-qPCR primers.

Names	Sequences
shMETTL3#1	GCAAGTATGTTCACTATGAAA
shMETTL3#2	GCTGCACTTCAGACGAATTAT
shH19#1	GAGTTAGCAAAGGTGACATCT
shH19#2	GCTCTGGAAGGTGAAGCTAGA
shH19#3	CCTGGGCCTTTGAATCCGGCCACAAAACC
siH19#1	GAATATGCTGCACTTTACA
siH19#2	TCACCTTTGCTAACTCTCC
siYTHDF1	TTCATGAACAACTAGACGC
siYTHDF2	TTGCTTGCAACTTCTGTGC
siYTHDF3	TTTAGGTCTCTGATCCACG
siIGF2BP1#1	TACTGTACCATACTGAGCC

siIGF2BP1#2	TTGTAATTCTACTTTCCCG
siIGF2BP1#3	TTGCTCACAGTTCTCTACT
siIGF2BP2	TAAAGCTTGTTCATCATCC
SiIGF2BP3	TAAACTATCCAGCACCTCC
siPPARγ#1	CATTCCATTCACAAGAACA
siPPARγ#2	GAGAAGATAAAATCAAGTT
qPCR-METTL3-F	TTGTCTCCAACCTTCCGTAGT
qPCR-METTL3-R	CCAGATCAGAGAGGTGGTGTAG
qPCR-E-cadherin-F	CGAGAGCTACACGTTCACGG
qPCR-E-cadherin-R	GGGTGTCGAGGGAAAAATAGG
qPCR-N-cadherin-F	TCAGGCGTCTGTAGAGGCTT
qPCR-N-cadherin-R	ATGCACATCCTTCGATAAGACTG
qPCR-Vimentin-F	AAGGTGAACCAGAGAGTAAGACG
qPCR-Vimentin-R	CGGTGTCGGTACTTTTTGTCC
qPCR-GAPDH-F	GGAGCGAGATCCCTCCAAAAT
qPCR-GAPDH-R	GGCTGTTGTCATACTTCTCATGG
qPCR-MMP2-F	AGTGACGGAAAGATGTGGTGT
qPCR-MMP2-R	CTTGGTGTAGGTGTAAATGGG
qPCR-MMP9-F	AGACCTGGGCAGATTCCAAAC
qPCR-MMP9-R	CGGCAAGTCTTCCGAGTAGT
qPCR-ICAM1-F	ATGCCCAGACATCTGTGTCC
qPCR-ICAM1-R	GGGGTCTCTATGCCCAACAA
qPCR-H19-F	TGCTGCACTTTACAACCACTG
qPCR-H19-R	ATGGTGTCTTTGATGTTGGGC
qPCR-YTHDF1-F	ACCTGTCCAGCTATTACCCG
qPCR-YTHDF1-R	TGGTGAGGTATGGAATCGGAG
qPCR-YTHDF2-F	GTTGGTAGCGGGTCCATTACT
qPCR-YTHDF2-R	GGTCTTCAGTTTAGGTTGCTGT
qPCR-YTHDF3-F	TCAGAGTAACAGCTATCCACCA
qPCR-YTHDF3-R	GGTTGTCAGATATGGCATAGGCT
qPCR-IGF2BP1-F	GGCCATCGAGAATTGTTGCAG
qPCR-IGF2BP1-R	CCAGGGATCAGGTGAGACTG
qPCR-IGF2BP2-F	CCGCAGCGGGAAATCAATCT
qPCR-IGF2BP2-R	ACGAAATATCCCGCCTCATTTAC
qPCR-IGF2BP3-F	ACGAAATATCCCGCCTCATTTAC
qPCR-IGF2BP3-R	GCAGTTTCCGAGTCAGTGTTCA
qPCR-PPARα-F	TTCGCAATCCATCGGCGAG
qPCR-PPARα-R	CCACAGGATAAGTCACCGAGG
qPCR-PPARβ-F	CAGGGCTGACTGCAAACGA
qPCR-PPARβ-R	CTGCCACAATGTCTCGATGTC
qPCR-PPARγ-F	ACCAAAGTGCAATCAAAGTGGA
qPCR-PPARγ-R	ATGAGGGAGTTGGAAGGCTCT
MeRIP-qPCR-H19-F	ACATGAAAGAAATGGTGCTA
MeRIP-qPCR-H19-R	CGATTCCTGAGTCAGGTAGT

qPCR-Hexon-F	ATGGTCGCTATGTGCCCTTC
qPCR-Hexon-R	CTGGCTCCGTCAACCCTTAG

Supplementary Table 3. Antibodies Information.

**Antibody name**	**Source**	**Identifier**
METTL3	ABclonal	A8370
E-cadherin	CST	3195S
N-cadherin	CST	13116S
Vimentin	CST	5741S
IGF2BP1	ABclonal	A13581
IGF2BP2	ABclonal	A14103
IGF2BP3	ABclonal	A6099
YTHDF1	ABclonal	A13260
YTHDF2	ABclonal	A15616
YTHDF3	ABclonal	A8395
PPARα	HUABIO	EM1707-71
PPARβ	HUABIO	ER1902-24
PPARγ	ABclonal	A11183
E1A	Abcam	ab204123
GAPDH	HUABIO	SA30-01
Mouse Control IgG	ABclonal	AC011
HRP Conjugated Goat anti-Mouse IgG polyclonal Antibody	HUABIO	HA1006
HRP Conjugated Goat anti-Rabbit IgG polyclonal Antibody	HUABIO	HA1001
Ki67	ABclonal	A20018

Supplementary Table 4. m6A sites information on H19 sequence.

**#**	**Position**	**Sequence context**	**Score(combined)**	**Decision**
1	767	GACAG GGACA UGGCA	0.598	m6A site (Moderate confidence)
2	779	GCAGG GGACA CAGGA	0.570	m6A site (Moderate confidence)
3	786	ACACA GGACA GAGGG	0.622	m6A site (High confidence)
4	907	CGGGA AGACA GGCAG	0.541	m6A site (Low confidence)
5	980	CCCCG GGACA UUGCG	0.646	m6A site (High confidence)
6	1078	ACAGU GGACU UGGUG	0.663	m6A site (High confidence)
7	1438	AAGCA GGACA UGACA	0.611	m6A site (High confidence)
8	1467	GGCGA GGACA GAGGA	0.593	m6A site (Moderate confidence)
9	1591	UCCCA GAACC CACAA	0.554	m6A site (Low confidence)
10	1648	AAUCC GGACA CAAAA	0.706	m6A site (Very high confidence)
11	1708	CUACC UGACU CAGGA	0.587	m6A site (Moderate confidence)
12	1744	UAGAG GAACC AGACC	0.553	m6A site (Low confidence)
13	1772	AUCAA AGACA CCAUC	0.629	m6A site (High confidence)
14	1783	CAUCG GAACA GCAGC	0.619	m6A site (High confidence)

15	2170	CCGGG UGACU GGGCG	0.538	m6A site (Low confidence)
16	2319	GCCCU GGACU CAUCA	0.673	m6A site (Very high confidence)

Figure S1: METTL3 regulates the expression of migration-related proteins.

A and B. The mRNA and protein expression of E-cadherin, N-cadherin and Vimentin were determined by RT-qPCR (A) and western blot (B) in HuCC-T1 and RBE cells with METTL3 overexpression, respectively.

C. The mRNA expression of MMP2, MMP9 and ICAM1 was determined by RT-qPCR in HuCC-T1 and RBE cells with METTL3 overexpression.

D and E. The mRNA and protein expression of E-cadherin, N-cadherin and Vimentin were determined by RT-qPCR (D) and western blot (E) in HuCC-T1 and RBE cells with METTL3 knockdown, respectively.

F. The mRNA expression of MMP2, MMP9 and ICAM1 was determined by RT-qPCR in HuCC-T1 and RBE cells with METTL3 knockdown.

G. The correlation between the expression of METTL3 and the indicated factors using GSE33327 database (n=149).

The results are presented as mean ± SD of three independent experiments. *P < 0.05, **P < 0.01, ***P < 0.001, according to a Student's t test.

Figure S2: H19 affects the proliferation and migration of ICCA cells.

A. The proliferation of HuCC-T1 and RBE cells with H19 overexpression was measured

by colony formation assay.

B. The migration of HuCC-T1 and RBE cells with H19 overexpression was measured by wound healing assay.

C. The proliferation of HuCC-T1 and RBE cells with H19 knockdown was measured by colony formation assay.

D. The migration of HuCC-T1 and RBE cells with H19 knockdown was measured by wound healing assay.

E. The protein expression of E-cadherin and N-cadherin was determined by western blot in HuCC-T1 and RBE cells with H19 overexpression or knockdown.

The results are presented as mean ± SD of three independent experiments. $^{*}P < 0.05$, $^{**}P < 0.01$, $^{***}P < 0.001$, according to a Student's t test. Scale bar, 100 μm.

Figure S3: The effect of METTL3 on cell migration and H19 stability.

A. The migration of RBE cells with METTL3 mutants overexpression was measured by transwell assay.

B. The migration of RBE cells with METTL3 mutants overexpression and H19 overexpression was measured by by transwell assay.

C and D. The H19 RNA decay rate was determined in METTL3-overexpressed (C) or

knockdown (D) HuCC-T1 and RBE cells after treatment with actinomycin D.

The results are presented as mean ± SD of three independent experiments. $^{**}P < 0.01$, $^{***}P < 0.001$, according to a Student's t test. Scale bar, 100 μm.

Figure S4: Downregulation of IGF2BP1 reduces the stability of H19.

A. The mRNA expression of reader YTHDFs and IGF2BPs was determined by RT-qPCR in HuCC-T1 cell with respective siRNAs transfection.

B. H19 level was determined by RT-qPCR in the indicated YTHDFs- or IGF2BPs-knockdown HuCC-T1 cells after treatment with actinomycin D.

C. The interaction between H19 and the indicated reader proteins in HuCC-T1 cells was verified by RIP-qPCR.

D. The correlation between the expression of H19 and the indicated reader proteins in GEPIA dataset.

E. The downregulated mRNA level of IGF2BP1 in three respective siRNA-treated HuCC-T1 and RBE cells was confirmed by RT-qPCR.

F. H19 expression was determined by RT-qPCR in HuCC-T1 and RBE cells with IGF2BP1 knockdown.

G. The interaction between IGF2BP1 and H19 was verified by RIP-qPCR.

H. The H19 RNA decay rate was determined in IGF2BP1 knockdown or IGF2BP1 knockdown and METTL3-overexpressed HuCC-T1 and RBE cells after treatment with actinomycin D.

The results are presented as mean ± SD of three independent experiments. *P < 0.05, **P < 0.01, ***P < 0.001, ns, no significance, according to a Student's t test.

Figure S5: The expression of PPARγ is regulated by METTL3 and H19.

A. KEGG analysis terms of the 34 genes which expression are correlated with H19 expression in GSE33327 ICCA dataset (n=149).

B and C. RT-qPCR analysis was performed to detect PPARα, PPARβ and PPARγ mRNA expression in HuCC-T1 and RBE cells with H19 overexpression (B) or knockdown (C), respectively.

D. PPARγ mRNA expression was downregulated by siRNAs treatment in HuCC-T1 cells.

E. PPARγ mRNA expression was determined by RT-qPCR in HuCC-T1 cell with the indicated siRNAs treatment.

F and G. RT-qPCR analysis was performed to detect PPARα, PPARβ and PPARγ mRNA expression in HuCC-T1 and RBE cells with METTL3 overexpression (F) or knockdown (G).

H. PPARγ mRNA expression was determined by RT-qPCR in HuCC-T1 cell with METTL3 overexpression and the indicated siRNAs treatment.

I. The correlation between the expression of METTL3 and PPARα, PPARβ, PPARγ in GSE33327 dataset (n=149).

J. Kaplan–Meier survival analysis of the correlation between PPARγ expression and overall survival in the TJ cohort.

K. The correlation between H19 and PPARγ expression in the TJ cohort.

L. The correlation between METTL3 and PPARγ expression in the TJ cohort.

The results are presented as mean ± SD of three independent experiments. *P < 0.05, **P < 0.01, ***P < 0.001, ns, no significance, according to a Student's t test.

Figure S6: Overview of the oncolytic adenovirus, IC_{50} assay, and animal experiment design.

A. The schematic overview of the structure of the constructed oncolytic adenovirus. ITR, inverted terminal repeat.

B. IC_{50} determination of BAY-4931 in HuCC-T1 and RBE cells.

C. The schematic overview of animal experiment. HuCC-T1 cells were subcutaneously inoculated into NOD/SCID mice at 1×10^7 cells per mouse. When tumor volume

reached approximately 100-120 mm^3 (Day0), the mice were randomly divided into four groups (n=8) and treated with 5×10^8 plaque forming unit (PFU) SD55-EGFP, SD55-H19 or SD55-H19 plus BAY-4931 (30mg/kg, gavage) once a day for repeated four times. PBS and DMSO treatment were used as control. s.c., subcutaneous, i.t., intratumoral injection, i.g., intragastric gavage.

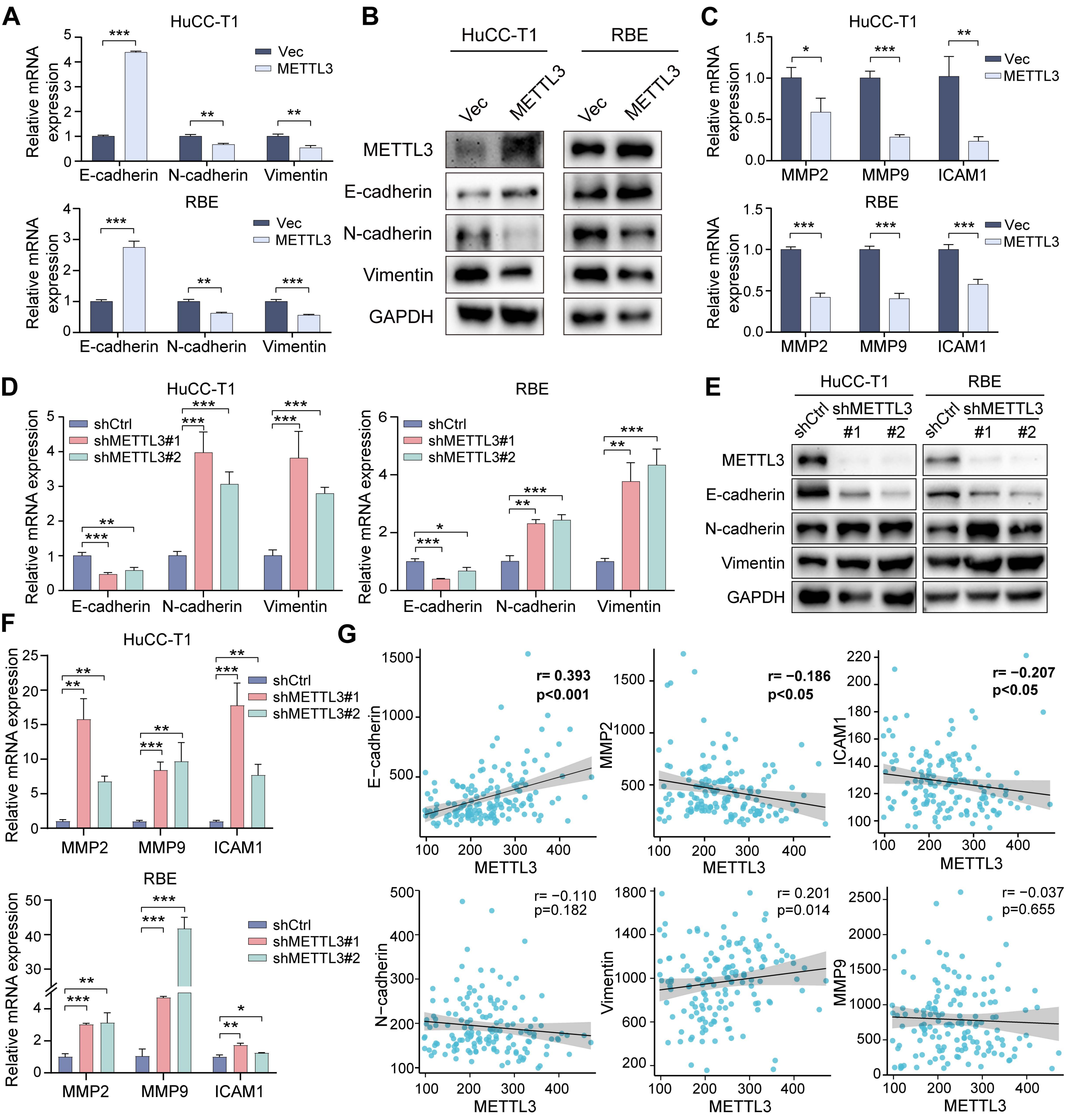
A
HuCC-T1
Relative mRNA expression
Vec
METTL3
E-cadherin
N-cadherin
Vimentin
RBE
B
HuCC-T1
RBE
Vec
METTL3
METTL3
E-cadherin
N-cadherin
Vimentin
GAPDH
C
HuCC-T1
MMP2
MMP9
ICAM1
RBE
D
shCtrl
shMETTL3#1
shMETTL3#2
E
shMETTL3
#1
#2
F
G
r= 0.393
p<0.001
r= −0.186
p<0.05
r= −0.207
p<0.05
r= −0.110
p=0.182
r= 0.201
p=0.014
r= −0.037
p=0.655

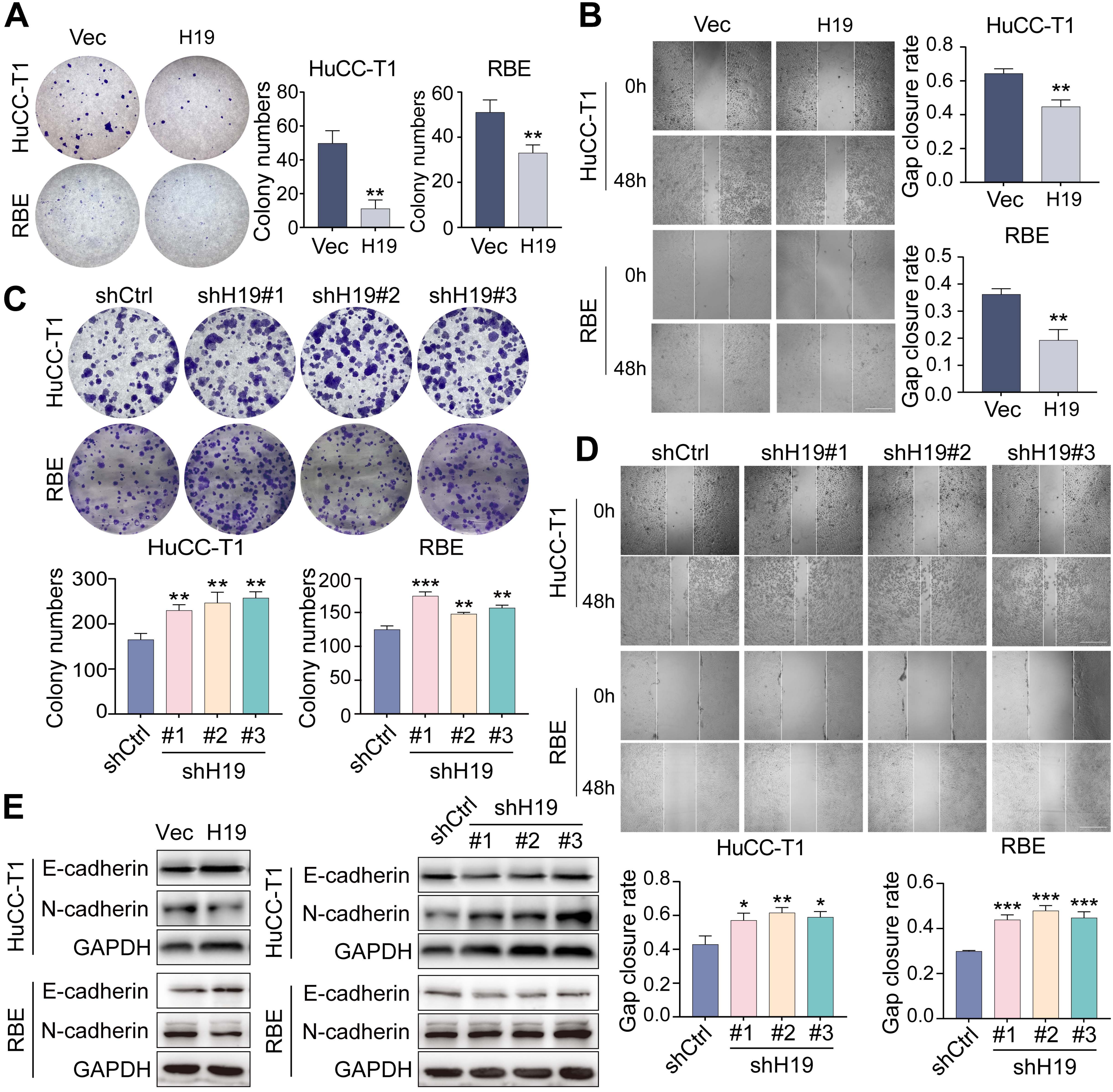

A
Vec
H19
HuCC-T1
RBE
Colony numbers
Vec
H19
**
B
Vec
H19
0h
48h
HuCC-T1
RBE
Gap closure rate
Vec
H19
**
C
shCtrl
shH19#1
shH19#2
shH19#3
HuCC-T1
RBE
Colony numbers
shCtrl
#1
#2
#3
shH19
**
***
D
shCtrl
shH19#1
shH19#2
shH19#3
0h
48h
HuCC-T1
RBE
Gap closure rate
shCtrl
#1
#2
#3
shH19
*
**
***
E
Vec
H19
shCtrl
shH19
#1
#2
#3
HuCC-T1
RBE
E-cadherin
N-cadherin
GAPDH

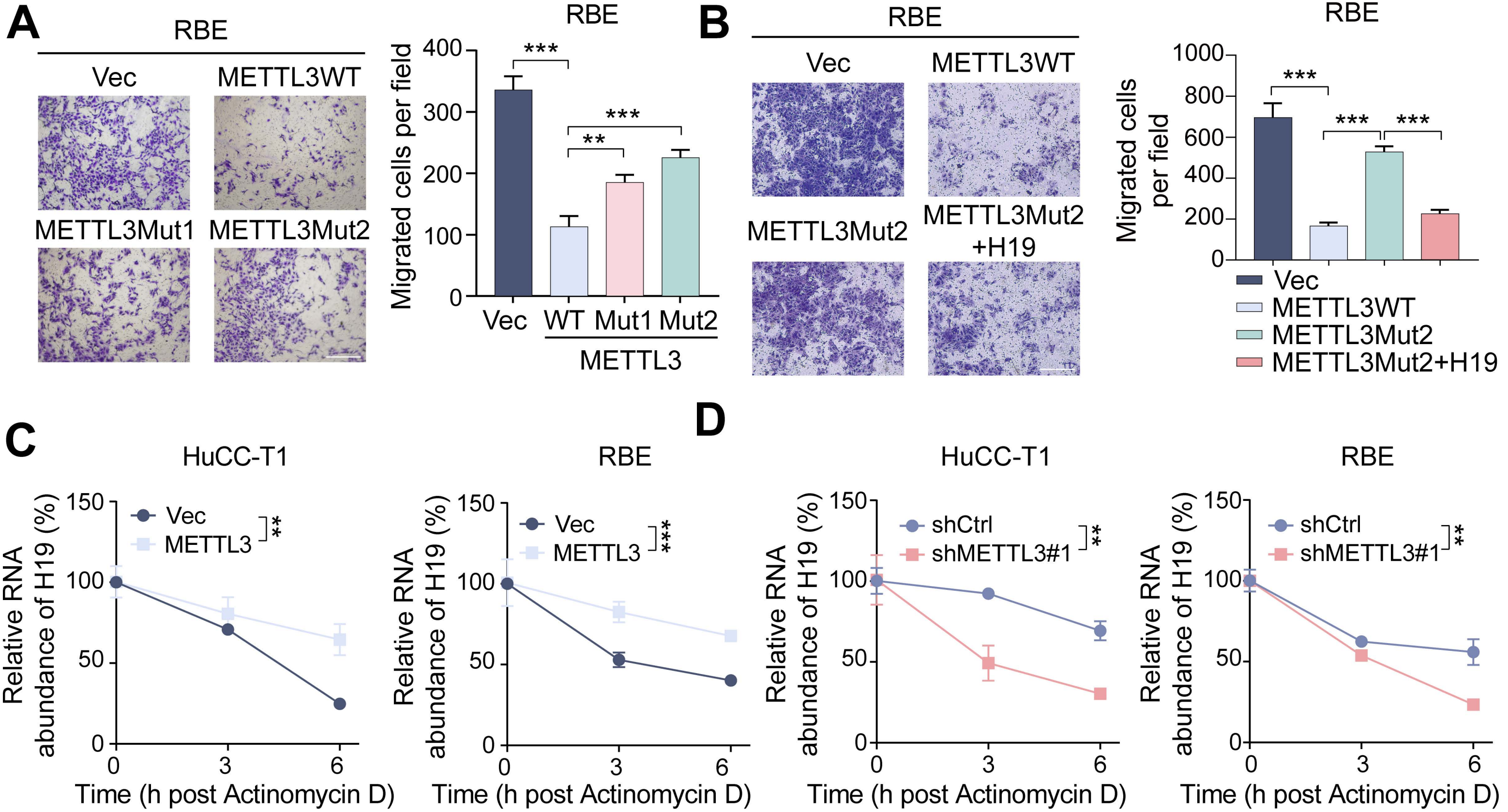

A
RBE
Vec
METTL3WT
METTL3Mut1
METTL3Mut2
Migrated cells per field
Vec
WT
Mut1
Mut2
METTL3
B
RBE
Vec
METTL3WT
METTL3Mut2
METTL3Mut2 +H19
Migrated cells per field
METTL3Mut2+H19
C
HuCC-T1
RBE
Relative RNA abundance of H19 (%)
Vec
METTL3
Time (h post Actinomycin D)
D
HuCC-T1
RBE
shCtrl
shMETTL3#1

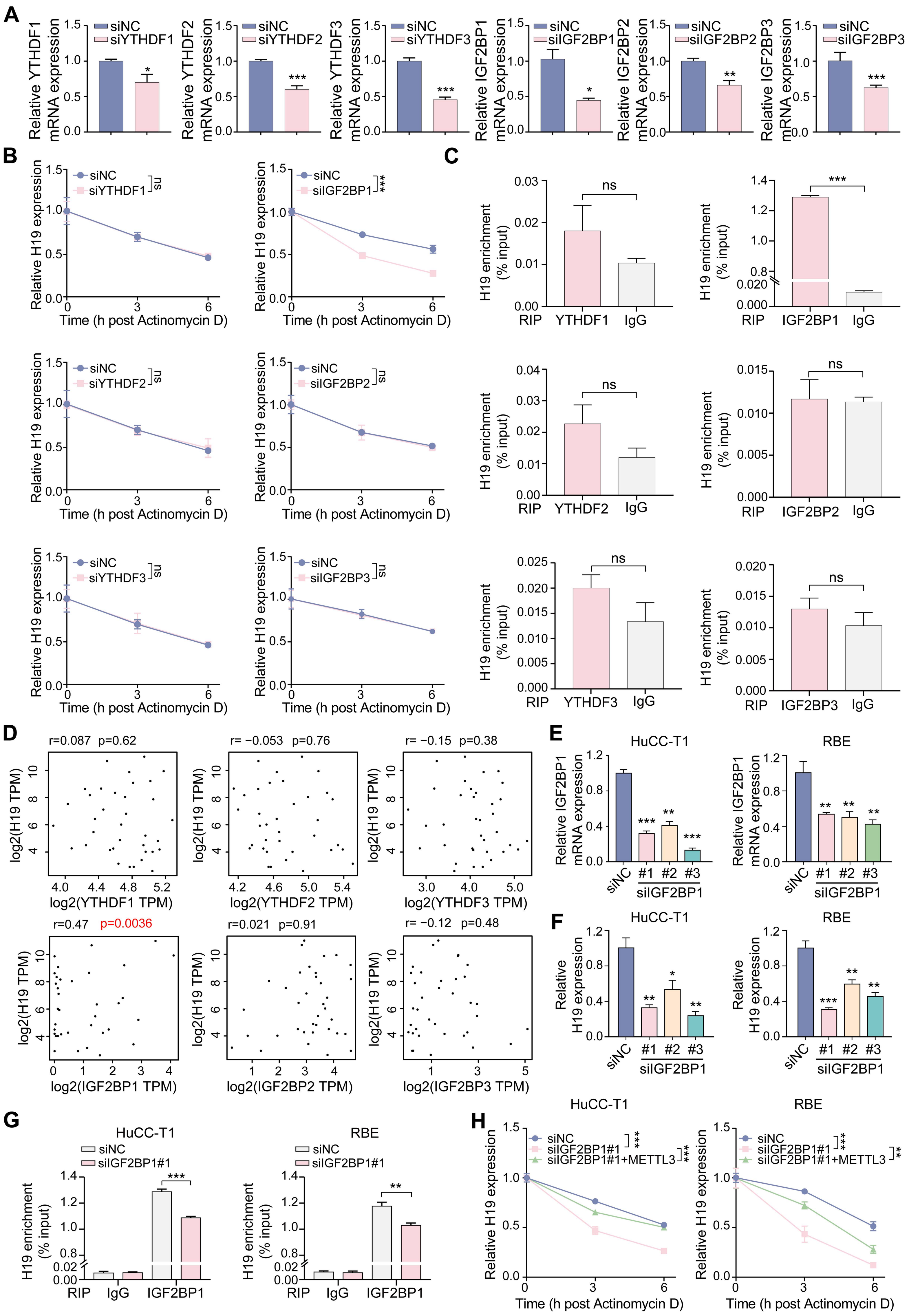

A
siNC
siYTHDF1
siYTHDF2
siYTHDF3
siIGF2BP1
siIGF2BP2
siIGF2BP3
Relative YTHDF1 mRNA expression
Relative YTHDF2 mRNA expression
Relative YTHDF3 mRNA expression
Relative IGF2BP1 mRNA expression
Relative IGF2BP2 mRNA expression
Relative IGF2BP3 mRNA expression
B
Relative H19 expression
Time (h post Actinomycin D)
ns
***
C
H19 enrichment (% input)
RIP YTHDF1 IgG
RIP IGF2BP1 IgG
RIP YTHDF2 IgG
RIP IGF2BP2 IgG
RIP YTHDF3 IgG
RIP IGF2BP3 IgG
D
r=0.087 p=0.62
r= −0.053 p=0.76
r= −0.15 p=0.38
r=0.47 p=0.0036
r=0.021 p=0.91
r= −0.12 p=0.48
log2(H19 TPM)
log2(YTHDF1 TPM)
log2(YTHDF2 TPM)
log2(YTHDF3 TPM)
log2(IGF2BP1 TPM)
log2(IGF2BP2 TPM)
log2(IGF2BP3 TPM)
E
HuCC-T1
RBE
Relative IGF2BP1 mRNA expression
siNC #1 #2 #3
siIGF2BP1
F
Relative H19 expression
G
siNC
siIGF2BP1#1
RIP IgG IGF2BP1
H
siNC
siIGF2BP1#1
siIGF2BP1#1+METTL3

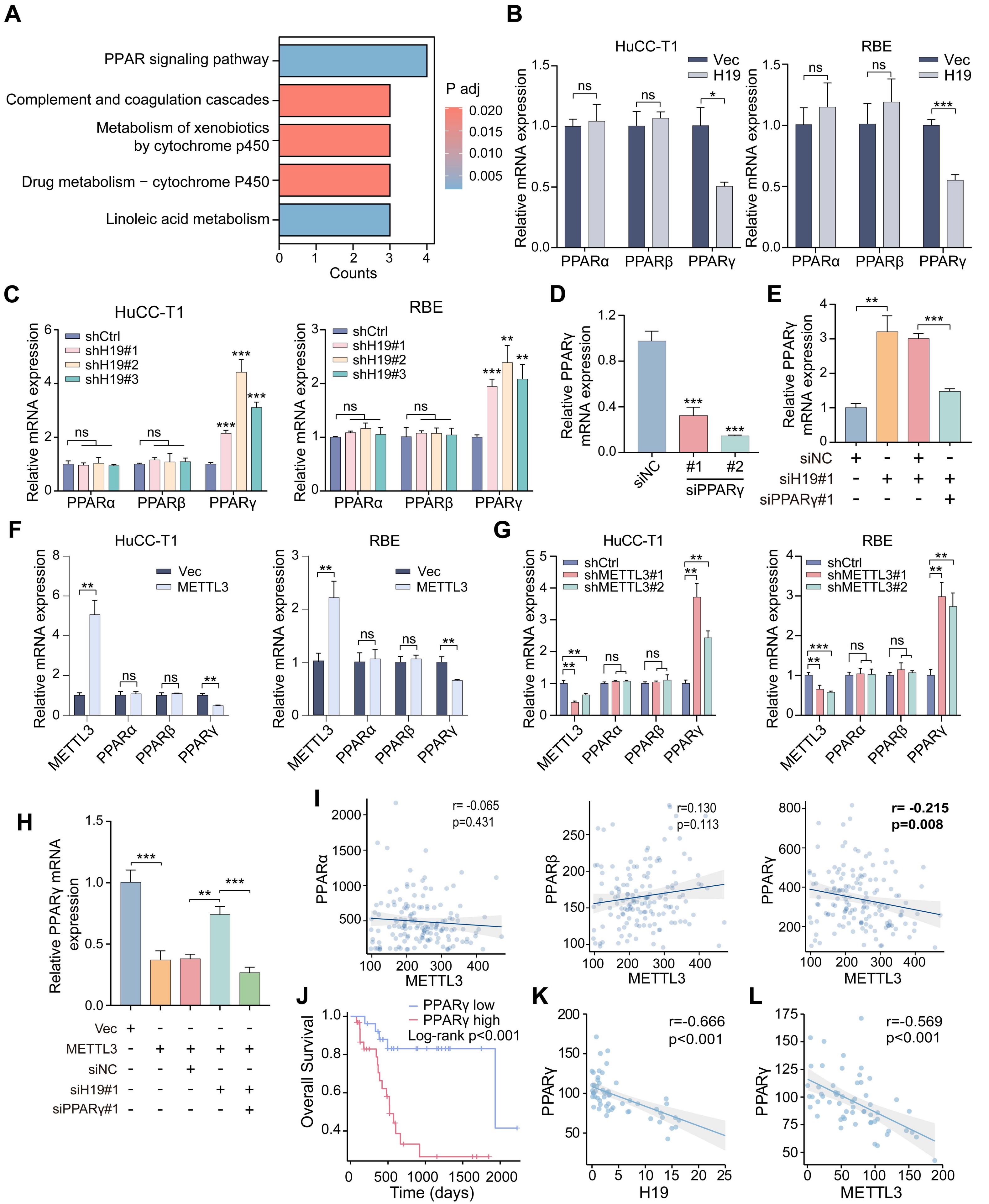

A
PPAR signaling pathway
Complement and coagulation cascades
Metabolism of xenobiotics by cytochrome p450
Drug metabolism − cytochrome P450
Linoleic acid metabolism
Counts
P adj
B
HuCC-T1
RBE
Vec
H19
Relative mRNA expression
PPARα
PPARβ
PPARγ
C
shCtrl
shH19#1
shH19#2
shH19#3
D
Relative PPARγ mRNA expression
siNC
siPPARγ
E
Relative PPARγ mRNA expression
siNC
siH19#1
siPPARγ#1
F
METTL3
G
shMETTL3#1
shMETTL3#2
H
Relative PPARγ mRNA expression
I
r= -0.065
p=0.431
r=0.130
p=0.113
r= -0.215
p=0.008
J
PPARγ low
PPARγ high
Log-rank p<0.001
Overall Survival
Time (days)
K
r=-0.666
p<0.001
H19
L
r=-0.569
p<0.001

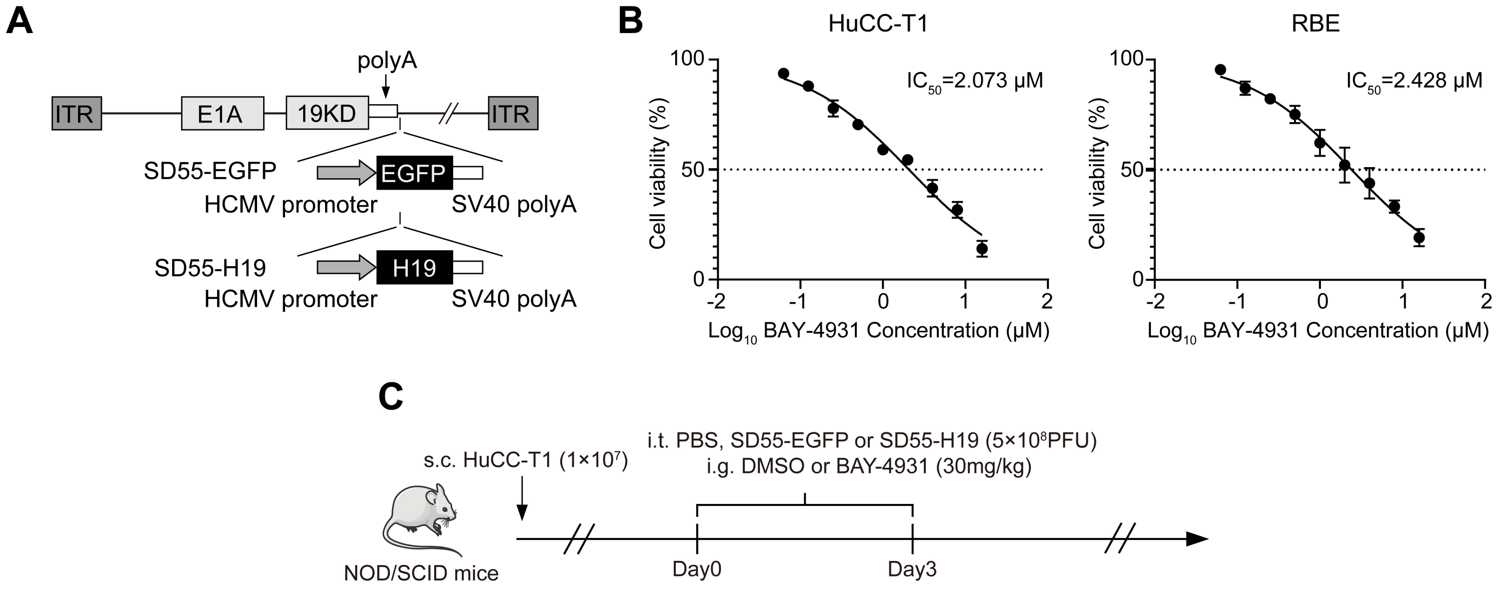
A
polyA
ITR
E1A
19KD
ITR
SD55-EGFP
EGFP
HCMV promoter
SV40 polyA
SD55-H19
H19
HCMV promoter
SV40 polyA
B
HuCC-T1
IC50=2.073 μM
Cell viability (%)
Log10 BAY-4931 Concentration (μM)
RBE
IC50=2.428 μM
Cell viability (%)
Log10 BAY-4931 Concentration (μM)
C
s.c. HuCC-T1 (1×10^7)
i.t. PBS, SD55-EGFP or SD55-H19 (5×10^8PFU)
i.g. DMSO or BAY-4931 (30mg/kg)
NOD/SCID mice
Day0
Day3
